# Innovative Didactic Learning Formats: Have They Improved Dental Education? A Systematic Review and Meta‐Analysis

**DOI:** 10.1111/iej.70006

**Published:** 2025-08-28

**Authors:** Ana Arias, Min‐Seock Seo, Lucia Gancedo‐Caravia, Isabel Fernandez‐Garcia, Juan José Pérez‐Higueras

**Affiliations:** ^1^ Department of Conservative and Prosthetic Dentistry School of Dentistry, Complutense University Madrid Spain; ^2^ Department of Conservative Dentistry Wonkwang University Daejeon Dental Hospital Daejeon Korea

**Keywords:** blended learning, dental education, educational outcome, innovative teaching methods, student satisfaction, traditional learning

## Abstract

**Background:**

New learning methods require higher professor‐to‐student ratios, increased faculty preparation time, continuous professional development for educators, and expanded physical spaces within university settings.

**Objectives:**

This systematic review aimed to answer the following PICO question: In dental students (P), what is the effectiveness of innovative formats of learning (I) in comparison with traditional formats (C) in terms of educational outcomes and satisfaction (O)?

**Methods:**

After PROSPERO protocol registration, a literature search was conducted using Web of Science (WoS), Scopus, PubMed and Cochrane Central Register of Controlled Trials. Selection of studies was performed in a three‐step process: identification, screening and eligibility. Data was extracted and analysed qualitatively and quantitatively. A random‐effects meta‐analysis was conducted to provide an estimate of the effect of innovative teaching formats in dental education. Additionally, subgroup analyses were performed to investigate potential differences in effectiveness based on the type of innovative teaching intervention.

**Results:**

One hundred and nineteen studies matched the inclusion criteria and were included in the systematic review. A meta‐analysis of 23 studies (1074 students in the control and 1021 in the experimental group) revealed significant differences in favour of innovative teaching methods (*p* < 0.00001) with considerable heterogeneity (*χ* ^2^ = 297.46, *p* < 0.00001; *I* ^2^ = 93%). Subgroup analysis also revealed significantly different results depending on the innovative teaching approach (*p* = 0.02). Both asynchronous independent learning and synchronous learning, either in a large group with the whole class of students using blended learning or in small groups, resulted in a significantly better outcome than traditional learning (overall effect: *Z* = 5.85; *p* < 0.00001); however, synchronous blended learning showed a significantly better outcome than the rest of the subgroups (mean difference = 16.59; 95% CI = 9.03–24.15). The quality of the studies varied, with some facing methodological challenges such as inconsistent outcome measurement, which can impact the generalisability of the findings.

**Conclusions:**

Innovative strategies lead to superior knowledge acquisition in comparison with traditional methods. Subgroup analyses favoured synchronous blended learning, but both asynchronous independent learning and synchronous learning formats, whether implemented in large‐group settings via blended approaches or in small‐group environments, are more effective than traditional instruction.

**Trial Registration:**

PROSPERO (CRD42024569691)

## 1 Introduction

Dental education has traditionally relied on a didactic approach primarily focused on teaching from instructor to student, often in lecture formats (De Moor et al. 2013). However, this traditional method presents several challenges for students. As they are required to absorb large amounts of information and keep up with the rapidly evolving field of dentistry, the task becomes increasingly difficult. With the rise of technology and the growing use of tech‐based learning methods, traditional teaching methods may no longer be the most effective resource (Alrahlah 2016). Thus, there is a growing need for innovative approaches to educational delivery. Many new educational models have emerged over the past 50 years and have attempted to harmonise teaching and learning based on the principle that students should drive their own learning process (Trullàs et al. 2022).

Problem‐based learning (PBL) was first introduced into dental education in the 1990s at the Faculty of Odontology in Malmö, Sweden (Rohlin et al. 1998). The core model of PBL consists of the following six characteristics: Learning is student‐centred; learning occurs in small groups of students; the teacher is a facilitator or guide; problems form the organising focus and stimulus for learning; problems are a vehicle for the development of problem‐solving skills; and new information is attained through self‐directed learning (Barrows 1996).

However, despite the advantages of this method, its implementation requires a relatively large number of facilitators and multiple small spaces to accommodate groups, posing logistical challenges for educational institutions (Winning and Townsend 2007). Furthermore, standardisation of training quality may be problematic due to differences in the ability of small groups to interpret clinical cases and appropriately identify intended learning objectives (Burgess et al. 2017).

To address some drawbacks of PBL, attention has been drawn to an alternative learner‐centred teaching model known as team‐based learning (TBL) (Michaelsen and Sweet 2008). TBL involves large group classes (which can be several hundred students) divided into smaller teams (typically 5–7), together with a smaller number of facilitators (typically 2–3) (Parmelee et al. 2012).

Several studies have shown favourable outcomes for blended learning approaches, such as the flipped classroom (FC) (Xiao et al. 2018; Wang et al. 2021). The FC approach ‘flips’ the traditional classroom. Instead of attending didactic lectures for knowledge acquisition followed by independent assignments/homework, the learner performs independent, self‐directed didactic learning for knowledge acquisition followed by classroom‐based discussion or debates. Learner‐centric discussions facilitated by an educator help create learning communities and enable peer‐to‐peer training, dialogue and support (Young et al. 2014).

Active learning as team‐based or blended methodologies is based on the commonly used learning theories—constructivism, cognitive and social connectivism—and the accumulated practical experience in education (Garrison and Kanuka 2004). These educational approaches actively engage students in the learning process through hands‐on activities, collaborative projects and technology‐enhanced environments. In recent years, interactive technologies have enabled applications with increasingly advanced personalisation features to keep students engaged (Abykanova et al. 2016).

Active student participation in the learning process using serious games is also being incorporated into dental education. The use of trivia games (Felszeghy et al. 2019) or escape rooms (Sauze et al. 2024) has been shown to improve student engagement and optimise educational outcomes (Warsinsky et al. 2021).

In early 2020, the emergence of the COVID‐19 pandemic disrupted numerous industries, prompting significant and irreversible changes. One of the most profound impacts was the rapid adoption and evolution of online and virtual teaching methods (Proffitt 2020). Additionally, the pandemic accelerated the shift towards a more student‐centred approach, with teaching strategies increasingly tailored to individual learning styles, preferences and pacing. Driven by necessity, this paradigm shift has demonstrated long‐term advantages and sustainability, extending beyond the immediate challenges of the pandemic (Reyes‐Millán et al. 2023).

E‐learning encompasses a variety of approaches that differ in structure, delivery and learner interaction. Learning can occur individually or collaboratively and can be delivered either synchronously (in real‐time) or asynchronously (on‐demand). Asynchronous e‐learning allows learners to access materials at different times from when they were produced or shared, promoting flexibility and self‐paced study. In contrast, synchronous e‐learning requires real‐time participation, often involving live lectures, discussions, or virtual meetings. The distinction between individual and collaborative learning further shapes the educational experience, with individual learning emphasising self‐guided progress (Ruiz et al. 2006).

There is a growing body of research examining the impact of these teaching methods on dental education; however, the findings remain inconsistent (Lau et al. 2021; Qin et al. 2023; Zhong et al. 2023; Karaca et al. 2024). Moreover, these innovative approaches often require higher professor‐to‐student ratios, increased faculty preparation time, continuous professional development for educators and expanded physical spaces within university settings. Given these challenges, further research is needed to assess the actual effectiveness of these teaching methods in dental education, particularly in terms of knowledge acquisition and long‐term retention.

Considering all these factors, the objective of the present systematic review was to answer the following PICO question: In dental students (P), what is the effectiveness of innovative formats of learning (I) in comparison with traditional formats (C) in terms of educational outcomes and satisfaction (O)?

## 2 Methods

### 2.1 Protocol Registration and Guidelines

**FIGURE 1:**
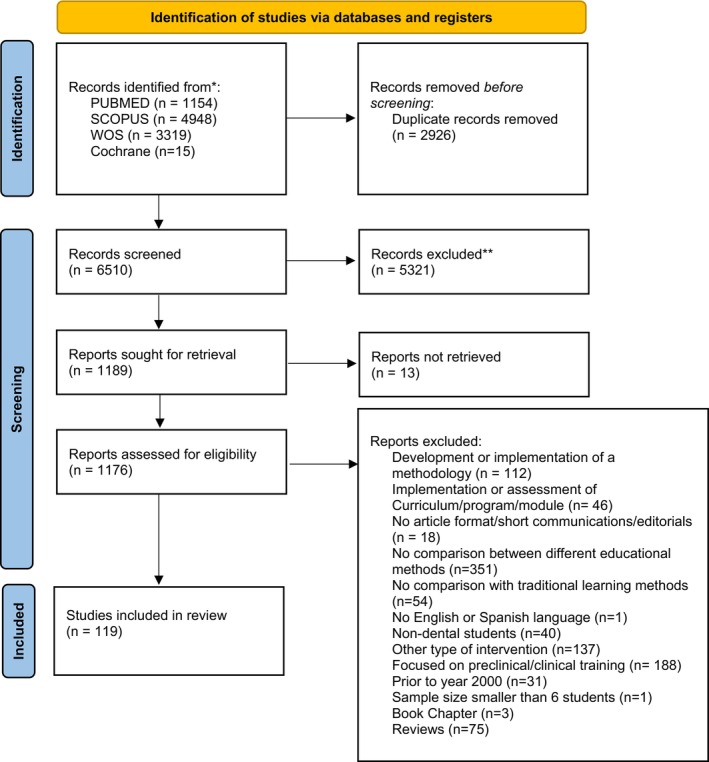
PRISMA 2020 flow diagram. *Consider, if feasible to do so, reporting the number of records identified from each database or register searched (rather than the total number across all databases/registers). **If automation tools were used, indicate how many records were excluded by a human and how many were excluded by automation tools. *Source:* Page et al. (2021).

The Preferred Reporting Items for Systematic Reviews and Meta‐Analysis (PRISMA) guidelines were followed (Page et al. 2021), and the protocol was registered in the International Prospective Register of Systematic Reviews PROSPERO (CRD42024569691). Figure 1 includes the PRISMA 2020 flow diagram.

### 2.2 Eligibility Criteria

- Population: Studies including dental students (undergraduate/postgraduate) or dental professionals.
- Intervention: studies including any form of active or interactive learning: small‐group discussion/gaming/collaborative learning/motivational learning/blended learning/peer learning/team‐based learning/debate/technology‐based learning/problem‐based learning/dynamic classroom/flipped classroom/seminar/role‐play/virtual reality/artificial intelligence/metaverse/social media/e‐learning.
- Comparison/control: studies including any form of traditional methods/lecture/large group teaching.
- Study design: Experimental studies with students (randomised control trials, comparative trials, non‐randomised), longitudinal observational studies (retrospective and prospective comparative cohort, case‐control and cross‐sectional studies).
- Outcome: Any form of knowledge acquisition or retention (learning outcome/academic performance/test scores) and satisfaction as an additional outcome.

### 2.3 Literature Search Strategy

After training and calibration by performing a literature search with a different number of keywords several times and obtaining comparable results, a comprehensive electronic search was conducted by two independent reviewers across PubMed, WoS, Scopus and Cochrane, from inception to date (2024 October 2). The search was restricted to studies published in English and Spanish. Most cited descriptors and keywords used in previous publications on the topic were incorporated into the electronic search strategy, using combined Medical Subject Heading (MeSH) terms and Boolean operators as follows: (“education” OR “educational” OR “educative” OR “teaching” OR “learning” OR “student” OR “instruction” OR “instructor” OR “supervisor”) AND (“small group” OR “seminars” OR “group work” OR “team‐based” OR “interactive” OR “active” OR “flipped classroom” OR “gamification” OR “gaming” OR “motivational” OR “collaborative” OR “blended classroom” OR “technology‐based” OR “debate” OR “group discussion” OR “problem‐based” OR “dynamic classroom” OR “peer learning” OR “artificial intelligence” OR “hands‐on” OR “role‐play” OR “virtual reality” OR “metaverse” OR “self‐learning” OR “social media” OR “e‐learning” OR “simulation” OR “traditional lecture” OR “lecture” OR “large group”) AND (“outcome” OR “efficiency” OR “effectiveness” OR “satisfaction” OR “skills” OR “treatment quality” OR “knowledge” OR “academic performance” OR “test score”) AND (“dental” OR “dentistry” OR “endodontics” OR “endodontology”).

Hand searches were also conducted in the reference lists of included papers and previously published reviews, as well as the last 10 years of *J Dental Edu*, *Eur J Dental Educ*, *JADE*, *J Dent Sci Educ*. To identify conference papers and other grey literature, an additional search was performed using Google Scholar and available repositories.

### 2.4 Study Selection

After executing the search strategy, the retrieved records were exported as Excel files and imported into the Rayyan AI‐Powered systematic review management platform mobile app (https://www.rayyan.ai). Upon upload, Rayyan automatically extracted titles and abstracts from the dataset. The automatic duplicate detection tool was activated to help reviewers find and remove duplicate records as a first screening; however, 10% of duplicates were not automatically detected and had to be manually reviewed and eliminated by the research team.

After duplicates were removed, the “BLIND ON” function was activated to ensure independent screening. Two reviewers independently assessed each study by selecting one of three options: *INCLUDE*, *EXCLUDE*, or *MAYBE*. After this initial screening phase, the “BLIND OF” option was then enabled to allow both reviewers to compare their selections. Any discrepancies (including all studies marked as *MAYBE* and those with discordant inclusion/exclusion decisions) were forwarded to a third reviewer for final adjudication.

Given the high number of initial search results and the uncertainty associated with using an app‐based screening process, only studies that did not include dental students or were non‐educational were excluded during this first phase, leaving 1189 articles still requiring screening that were exported for a second screening phase, which was manually conducted on computers. Due to the large volume of studies, the screening process was divided among multiple pairs of reviewers to ensure thorough assessment and minimise individual bias. This strategy was intended not only to improve efficiency but also to mitigate the risk of reviewer fatigue, which can affect decision‐making accuracy in large‐scale systematic reviews. The list of the excluded studies and reasons for exclusion from this second screening phase can be found as a Table S1.

Selection of relevant and appropriate studies was performed in a three‐step process: identification, screening and eligibility. The relevance of the articles was screened by titles, abstracts and the complete full text when necessary.

### 2.5 Data Extraction

The data extraction was performed by two reviewers who independently performed duplicate data extraction using a pre‐established and piloted spreadsheet. In the case of incomplete or missing data, the authors of the papers were contacted for clarification. In the case of non‐agreement between the reviewers, the data was discussed with a third reviewer. In the case of studies with multiple reports on the same study, the relevant data of interest was extracted.

The following details were extracted from the studies and included in a spreadsheet: name (first author) and year of publication, country (setting), ethics committee, outcome parameter, observation/type of assessment, study design, type of participants, topic thought, total number of participants and number of groups. For each group, the name of the group, the final number of students and results are facilitated. The better outcome and the significance are also registered. All extracted data is shown in Table 2.

### 2.6 Strategy for Data Synthesis

All data was analysed qualitatively and quantitatively, and a narrative synthesis of the included studies was performed. If the included studies were homogeneous, a quantitative meta‐analysis was considered using Review Manager (Review Manager (RevMan) [Computer program]) (The Cochrane Collaboration 2020). Data on the primary outcome were pooled and analysed using weighted mean differences and 95% confidence intervals (CI). Forest plots were created to illustrate the effects of the global estimation in the meta‐analysis and of the different subgroups. Statistical heterogeneity among studies was assessed with tau‐squared, the chi‐squared test for heterogeneity, the *I* ^2^ test and visual inspection of the forest plot.

### 2.7 Risk of Bias

The following items were independently evaluated by two reviewers for the critical appraisal of the included randomised controlled trials using the RoB version 2.0.: randomisation bias, bias due to deviations from intended interventions, bias due to missing outcome data, bias in measurement of the outcome, and bias in selection of the reported result. After assessing the five domains, an overall risk of bias (“low risk of bias”, “some concerns” or “high risk of bias”) was determined. Discrepancies were resolved by discussion and consensus. The study was considered at “low risk of bias” if only one out of the five domains was unclear; when two domains were unclear or one was at “high risk” the overall risk of bias was determined as “some concerns,” and the study was considered at “high risk of bias,” if one domain was at “high risk” and at least another showed “some concerns”. The ROBINS‐I tool (Risk Of Bias In Non‐randomised Studies of Interventions) was used to assess the risk of bias of non‐randomised studies of interventions. The tool considers seven domains of potential bias: confounding, participant selection, classification of interventions, deviations from intended interventions, missing data, measurement of outcomes and selection of the reported result. Each domain was evaluated independently by two reviewers and rated as low, moderate, serious, critical, or no information. Discrepancies were resolved by discussion and consensus. The overall risk of bias was determined by the highest risk level in any of the seven domains.

## 3 Results

### 3.1 Study Selection

Figure 1 shows the PRISMA flowchart of the literature search and the studies selected in this systematic review. According to the search strategy used, 9436 hits were extracted. After removing duplicates (*n* = 2926), 6510 studies were left for further screening. 5321 records were excluded, and 1189 were sought for retrieval. Thirteen could not be retrieved, and the corresponding author did not reply to the article request. Out of the remaining 1176, 1057 were excluded for a variety of reasons: Development or implementation of a methodology (112); implementation or assessment of curriculum/program/module (46); no article format/short communications/editorials (18); no comparison between different educational methods (351); no comparison with traditional learning methods (54); no English or Spanish (1); non‐dental students (40); Other type of interventions, such as no inclusion of any educational methodology, no strict comparison, or global evaluation of preferences (137); focused in preclinical/clinical training (165); previous to year 2000 (31); sample size smaller than 6 (1); book chapters (3); reviews (75). Grey literature was searched using Google Scholar, but no title or abstract matched the criteria.

### 3.2 Characteristics of the Included Studies and Synthesis of the Results

One hundred and nineteen studies matched the inclusion criteria and were included in the systematic review. Table 1 shows the characteristics of the 119 studies. As shown in Table 2, 58 out of the 119 were randomised controlled trials, and the rest responded to other types of study design. Most studies (*n* = 76) evaluated the knowledge acquisition of students when receiving traditional or innovative teaching methods; seven addressed the attitude, satisfaction, or perceptions of these methods by participants and 36 included both the analysis of the learning outcomes and the subjective component. As shown in the test group category, there is a wide range of innovative teaching strategies, the most common being FC and blended learning, self‐guided learning with or without additional material, problem‐based or case‐based learning, interactive synchronous sessions and learning based on games and different group performances in the classroom.

**TABLE 1:** Characteristics of the included studies.

Author, year (country)	Outcome parameter	Observation/type of assessment	Study design (ethics committe approval)	Topic taught	Participants: Type/Total *n*/No of groups	Control group		Test group		Aditional test group (s)		Better outcome	*p* (sig)
						Methodology (final *n*)	Results	Methodology (final *n*)	Results	Metodology (final *n*)	Results		
Al‐Ahmad 2010 (Jordan)	Attitude/Satisfaction/Perceptions	Questionnaire. 5‐point Likert scale	Cohorts comparison (Y)	Oral surgery	Final year DUS Graduates from 2 previous years/157/2.	Conventional teaching (85)	Mean (SD) (Ability to describe surgical details; Anatomy and instruments; Understanding of protocols; Dealing with complications; Resident duties and patient monitoring; Cross infection control; Risks; Falimiarity with surgical environment) = 3.30 (0.82); 2.78 (0.69); 2.46 (0.81); 2.66 (0.59); 2.54 (0.65); 2.96 (0.92); 2.13 (0.86); 2.78 (0.71)	Operating theater based learning (72)	Mean (SD) (Ability to describe surgical details; Anatomy and instruments; Understanding of protocols; Dealing with complications; Resident dutiesand patient monitoring; Cross infection control; Risks; Falimiarity with surgical environment) = 2.64 (0.90); 2.58 (0.73); 1.94 (0.66); 2.27 (0.64); 2.12 (0.64); 2.11 (0.86); 1.81 (0.61); 2.17 (0.82)			Test group (Operating theater) except for perceived knowledge of regional anatomy and familiarity with instruments	0.025–< 0.001
Alharbi 2020 (Saudi Arabia)	Knowledge acquisition	Improvement in learning comparing cores pre‐ and post‐intervention tests (Max. S: 20) and retention test after 10 weeks (Max. S: 5)	Cross over (Y)	Orthodontics	4th year DUS/34/2	Traditional lecture followed by questions during lecture with phone‐based audience response system (17)	Mean (SD) (Post‐test; Retention test) = 8.89 (3.89); 2.87 (1.51) Improvement of learning: 79.3%	Questions during lecture with phone‐based audience response system followed by traditional lecture (17)	Mean (SD) (Post‐test; Retention test) = 10 (2.74); 3.06 (1.49) Improvement of learning: 87.5%			—	
	Attitude/Satisfaction/Perceptions	Questionaire. 5‐point Likert scale					Overall level of satisfaction rates: Median and mode = 4		Overall level of satisfaction rates: Median and mode = 4			—	
Alharbi 2022 (Saudi Arabia)	Knowledge acquistion	Pre‐ and post‐intervention test scores. MCQ. (Max. S. not provided)	RCT (Y)	Orthodontics	4th year DUS/32/2	Virtual traditional learning with live video lectures (16)	Median = 9	Virtual flipped learning (recorded online lectures+virtual discussions) (17)	Median = 8.5			—	
	Attitude/Satisfaction/Perceptions	Rating questionnaire (Max. S. not provided)					Overall preference rating median = 5		Overall preference rating median = 9			Test group (virtual flipped learning)	< 0.001
Alhazmi 2020 (Saudi Arabia)	Knowledge acquistion	Test. Number of correct responses. (Max. S. not provided)	RCT (Y)	Orthodontics	Final year DUS/95/2	Lecture based learning (48)	Mean (SD) = 136.77 (35.19)	Case Based Learning (47)	Mean (SD) = 192.72 (41.31)			Test group (Case Based Learning)	< 0.001
	Attitude/Satisfaction/Perceptions	Questionaire. 5‐point Likert scale										Test group (Case Based Learning) (higer scores in satisfaction, linking knowledge with practicality, confidence, increase ability to present case reports, increase power of creativity)	0.01–< 0.001
Alkahtani 2021 (Saudi Arabia)	Attitude/Satisfaction/Perceptions	Satisfaction questionaire. 3‐point Likert scale	Cohorts comparison (Y)	Endodontics	3rd year DUS/66/2	Face‐to‐face lectures (previous cohort) (37)	Understanding: % Responses: 82.05% Satisfactory, 17.95% Neutral Memorizing: % Responses: 61.54% Satisfactory, 33.33% Neutral, 5.13% Unsatisfactory	Online synchronized lectures (39)	Understanding: % Responses: 92.31 Satisfactory, 7.69% neutral Memorizing: % Responses: 76.9 Satisfied, 20.5% neutral, 2.6 unsatisfactory	Online asynchronized lectures (39)	Understanding: % Responses: 76.93 Satisfactory, 17.95 Neutral, 5.12% Unsatisfactory Memorizing: % Responses: 66.67 Satisfactory, 23.08 Neutral, 10.25% Unsatisfactory	—	
	Knowledge acquistion	Summative examination test scores. MCQ. (Max. S. not provided)					Mean (SD) = 27.47 (2.57)	Online synchronized and asynchronized lectures (39)	Mean (SD) = 28.55 (2.66)			—	
Al‐Riyami 2010 (UK)	Knowledge acquistion	Test scores after 1st and 2nd teaching episode (Max. S. not provided)	Cross over (N)	Orthodontics	Graduate Orthodontic students/30/2	Conventional face‐to‐face seminar followed by Virtual Learning environment Moodle Tutorial (15)	1st episode; 2nd episode: Mean (CI 95%) = 14.27 (12.18–16.35); 20.93 (19.12–22.75)	Virtual Learning environment Moodle tutorial followed by conventional face‐to‐face seminar (15)	1st episode; 2nd episode: Mean (CI 95%) = 13.85 (10.37–17.36); 22 (19.79–24.21)			—	
	Attitude/Satisfaction/Perceptions	Questionnaire at the end of the study										Test group (Out of all students, 16 reccomend the Virtual Learning environment Moodle Tutorial method while 14 reccomend the face to face).	
Alsufyani 2023 (Saudi Arabia)	Knowledge acquistion	Test scores. Structures to identify. Max. S: 20	RCT (Y)	Radiology	1st year DUS/69/2	Conventional training (Lecture‐based) (35)	Mean (SD) responses correct: 11.66 (2.69)	Virtual reality (34)	Mean (SD) responses correct: 9.88 (2.25)			Control group (Lecture based)	0.004
Alwadei 2020 (USA)	Knowledge acquistion	Difference between Pre‐ and post‐intervention test scores	Cohorts comparison (Y)	Not specified	2nd year DUS/343/3	Traditional face‐to‐face learning (112)	Mean improvement (SD): 18.11 (13.56)	Formative adaptive learning platform (45)	Mean improvement (SD): 22.33 (11.82)	Summative adaptive learning platform (183)	Mean improvement (SD): 28.82 (14.89)	Test group (Formative Adaptive Learning Platform)	< 0.001
Aly 2004 (Belgium)	Knowledge acquistion	Pre‐ and post‐intervention test scores. MCQ. Number of correct answers. (Max. S. not provided)	Controlled study (N)	Orthodontics	Final year DUS/26/2	Standard lectures (11)	Mean (SD) (Pre‐test; Post‐test) = 48.6 (15.1); 79.2 (19.2)	Interactive multimedia package (Compluted assisted learning) (15)	Mean (SD) (Pre‐test; Post‐test) = 54.1 (10.9); 86.2 (27.3)			—	
Anyanwu 2014 (Nigeria)	Knowledge acquistion	Pre‐ and post‐intervention test scores. Presented as percentage	RCT (N)	Anatomy	2nd year medicine and DUS/79/2	Non game (34)	Mean (SD) (Pre‐test; Post‐test) = 51.4 (13.8); 54.5 (14.2)	Game group (45)	Mean (SD) (Pre‐test; Post‐test) = 56.3 (12.1); 62.2 (9.4)			Test group (Game group)	< 0.05
Ariana 2016 (Australia)	Knowledge acquistion	Final examination test scores. Presented as percentage.	Cohorts comparison (N)	Histopatology	2nd year DUS/194/2	Traditional learning (90)	Mean (SD): 85.43% (10.23)	Blended learnig with electronic material (104)	Mean (SD): 96.13 (5.73)			Test group (Blended learninig with electronic material)	< 0.05
	Attitude/Satisfaction/Perceptions	Questionnaire. 5‐point Likert scale					Mean (Engaged me; Teaching was effective) = 4.42; 4.73	Blended learnig with electronic material (104)	Mean (Engaged me; Teaching was effective) = 4.67; 4.71			Test group (Blended learninig with electronic material)	< 0.05
Arias 2016 (USA)	Knowledge acquistion	Test scores. MCQ and short open‐ended questions. (Max. S.: 5)	Controlled study (Y)	Endodontics	1st year DUS/134/2	Traditional lecture (68)	Mean (SD) = 4.3 (1.2)	Small group discusion (66)	Mean (SD) = 4.49 (1.11)			—	
Azeem 2018 (Pakistan)	Knowledge acquistion	Difference between Pre‐ and post‐intervention test scores	RCT (N)	Occlusion	Final year DUS/40/2	Traditional didactic lecture (20)	Mean (SD) = 2.2 (1.6)	MCQ integrated lecture (20)	Mean (SD) = 4,8 (1,7)			Test group (MCQ integrated lecture)	< 0.001
Bains 2011 (UK)	Knowledge acquistion	Test score after the intervention. MCQ. Max. S: 10 (Data provided as proportion of students with correct answers. Max: 100%)	RCT (Y)	Orthodontics	4th year DUS/157/4.	Face‐to‐face learning (teacher led tutorial) (36)	% of correct answers (Q1; Q2; Q3; Q4; Q5; Q6; Q7; Q8; Q9; Q10) = 83; 11; 69; 94; 97; 75; 78; 97; 67; 53	E‐learning (online tutorial without teacher) (22)	% of correct answers (Q1; Q2; Q3; Q4; Q5; Q6; Q7; Q8; Q9; Q10) = 68; 18; 77; 86; 73; 55; 41; 68; 45; 32	E‐learning followed by face‐to‐face (14) & Face‐to‐face followed by e‐learning (18)	E‐learning followed by face‐to‐face: % Correct answers (Q1; Q2; Q3; Q4; Q5; Q6; Q7; Q8; Q9; Q10) = 64; 14; 57; 93; 79; 86; 86; 71; 71; 86 Face‐to‐face followed by e‐learning: % Correct answers (Q1; Q2; Q3; Q4; Q5; Q6; Q7; Q8; Q9; Q10) = 89; 33; 78; 100; 83; 72; 94; 100; 94; 67	Control and Aditional groups (face‐to face, e‐learning followed by face‐to‐face and face‐to‐face followed by e‐learning) (only in questions 7‐10)	0.0005–0.0107
Bhandary 2019 (India)	Knowledge acquistion	Pre‐ and post‐intervention test scores. Max. S: 30	RCT (Y)	Not specified	1st year DUS/150/4.	No peer assisted learning. Low performance students (34)	Post‐test 1 (before cross‐over): Mean (SD) = 10 (3.5)	Peer assisted learning. Low performance students (34)	Post‐test 1 (before cross‐over): Mean (SD) = 18 (3.5)	Peer assisted learning. High performance students (34) & No peer assisted learning. High performance students (34)	Peer assisted learning. High performance students: Post‐test 1 (before cross‐over): Mean (SD) = 16 (3.5) No peer assisted learning. High performance students: Post‐test 1 (before cross‐over): Mean (SD) = 21 (3.5)	Aditional test group (Peer assisted learning in high performance students)	0.001
	Attitude/Satisfaction/Perceptions	Questionnaire. 5‐point Likert scale										Test and aditional groups (Peer assisted learning): useful (59%); help retain content (55%); enhance communication skills and self‐learning (53%)	
Boonmak 2022 (Thailand)	Knowledge acquistion	Test scores of immediate and 3‐month post‐intervention tests. MCQ. Max S.: 100%	RCT (Y)	Basic lfe support	5th year DUS/78/2	Didactic lecture (39)	Immediate: Mean (SD) = 74.8% (7.8) 3‐month: Mean (SD) = 65.2% (8.1)	Online learning with Moodle (39)	Immediate: Mean (SD) = 77.3% (8.6) 3‐month: Mean (SD) = 66.5% (7.9)			—	
	Attitude/Satisfaction/Perceptions	Questionnaire. 5‐point Likert scale							Satisfaction: 4.59 (0.55)			—	
Cardozo 2023 (Brazil)	Knowledge acquistion	Test scores of pre‐ and 2 post‐intervention (after 1st and 2nd methodology) tests. Max. S.: 10	Cohorts comparison (N)	Physiology	1st year DUS/103/2	Traditional theoretical lectures followed by educational game (51)	Post‐test 1: Mean (SD) = 6.96 (2.00) Post‐test 2: Mean (SD) = 9.02 (2.00)	Active methodology (reduced dialogic class, game, quiz) followed by theoretical lecture (52)	Post‐test 1: Mean (SD) = 8.89 (0.99) Post‐test 2: Mean (SD) = 8.94 (2.06)			Test group (Active methodology followed by theoretical lecture)	< 0.05
Chen 2023 (China)	Attitude/Satisfaction/Perceptions	Sum of scores in two questionnairesregarding motivation (5‐point Likert scale) and experience (10‐point Likert scale)	Cross over (N)	Orthodontics	4th year DUS/102/2	Traditional case analysis by Power Point followed by virtual reality (42)	Motivation (attention; relevance; confidence; satisfaction): Mean (SD) = 19.5 (3.49); 19.9 (2.77); 17.5 (2.77); 19.2 (3.15) Experience (useful; enjoyable; engaging; likely to recommend): Mean (SD) = 7.99 (1.32); 7.82 (1.47);7.73 (1.63); 7.66 (1.62)	Virtual reality followed by traditional case analysis (43)	Motivation (attention; relevance; confidence; satisfaction): Mean (SD) = 21.9 (2.34); 21.4 (2.19); 19.33 (2.66); 21.3 (2.36) Experience (useful; enjoyable; engaging; likely to recommend): Mean (SD) = 8.46 (1.12); 8.33 (1.15); 8.39 (1.25); 8.62 (1.14)			Test group (Virtual reality followed by traditional case analysis)	< 0.0001
Chen 2022 (USA)	Attitude/Satisfaction/Perceptions	Course evaluation questionnaire. 4‐point Likert scale	Cohorts comparison (Y)	Orthodontics	Predoctoral students/116/2	In person lectures (53)	Mean ratings: 3.2; 3.2; 3.0; 3.1; 3.3;3.3;3.3; 3.1	Case‐based group discussions and online asynchronous lectures (63)	Mean ratings: 3.2; 3.3; 3.0; 3.2; 3.3;3.3;3.5; 3.2;3.2; 3.0; 3.1			—	
Costa‐Silva 2018 (Brazil)	Knowledge acquistion	Content evaluation of final scientific report (% of reports including main scientific concepts) and overall course scores (Max. S.: 10)	Controlled study (Y)	Cell biology	DUS/64/2	No project‐based learning (laboratory class only) (37)	Overall course score: Mean (SD) = 7.2 (1.6)	Project‐based learning (27)	Overall course score: Mean (SD) = 7.8 (1.2)			—	
DeBate 2014 (USA)	Knowledge acquisition	Post‐intervention (end of semester) assessment scores. Max. S.: 7	RCT (N)	Prevention of eating disorders	DUS/229/2	Alternative approach (conventional) (36)	Mean (SD) (Role beliefs; Benefits/barriers; Perceived threat; Self efficacy; Knowledge of eating disorders and findings; skills‐based knowledge) = 2.36 (0.41); 0.83 (0.79);2.32 (0.39); 1.82 (0.45); 4.24 (0.82); 5.41 (1.27)	Interactive e‐learning (193)	Mean (SD) (Role beliefs; Benefits/barriers; Perceived threat; Self efficacy; Knowledge of eating disorders and findings; skills‐based knowledge) = 2.35 (0.48); 1.16 (0.80); 2.42 (0.39); 2.23 (0.47); 4.50 (1.18); 6.41 (1.95)			Test group (Interactive e‐learning) in benefits/barriers, self‐efficacy and skills‐based knowledge	< 0.05
Deepak Nallaswamy 2019 (India)	Knowledge acquisition	Scores at the end of the year. Max. S.: 200	Cohorts comparison (N)	Conservative dentistry and endodontics	Final year DUS/150/2	Conventional classroom (75)	Mean grade: 130.93 (9.12)	Flipped classroom (75)	150.35 (10.93)			Test group (Flipped classroom)	< 0.05
Echeto 2015 (USA)	Knowledge acquisition	Final examination grades and pass rate. Max. S.: 1. Pass/fail threshold: 0.72	Cohorts comparison (Y)	Prostesis	Senior dental students/157/2	Conventional (79)	Mean (SD) = 0.70 (0.092); Pass rate = 48.1%	Team based Learning (78)	Mean (SD) = 0.758 (0.083); Pass rate = 71.8%			Test group (Team based learning)	< 0.05
Ehsan 2020 (Pakistan)	Knowledge acquisition	Difference between Pre‐ and post‐intervention test scores. MCQ. Max. S.: 10	Controlled Trial (Y)	Orthodontics	Final year DUS/342/2	Faculty guided learning (traditional tutorials) (227)	Mean (SD) (Post‐test; change) = 51.65 (21.29); 7.49 (18.23)	Peer assisted learning (115)	Mean (SD) (Post‐test; change) = 62.43 (16.01); 2.30 (16.68)			Control group (Faculty guided learning)	0.001
Farah‐Franco 2021 (USA)	Knowledge acquisition	Mean course grades in preclinical courses (Max. S. not provided)	Cohorts comparison (Y)	Essentials of clinical dentistry. Comprehensive care clinical dentistry	Different levels DUS/277/2	Traditional lecture (2 cohorts) (139)	Mean (SD) (Cohort A; Cohort B) = 88.96 (6.97); 89.46 (2.19)	hybrid lecture free—active learning (2 cohorts) (138)	Mean (SD) (Cohort C; Cohort D) = 89.98 (3.08); 90.19 (7.08)			—	
Freda 2016 (USA)	Knowledge acquisition	Pass rate in summative assesment. Clinical case evaluation. Pass/fail threshold: F ( = critical errors on four cases)	Cohorts comparison (N)	Orthodontics	3rd year DUS/162/2	Traditional approach (162)	Pass rate (Cohort 1; Cohort 2) = 87.50%; 70.27%	Test‐enhanced (Formative assessment sessions) (176)	Pass rate (Cohort 1; Cohort 2) = 82.45%; 83.53%			—	
Fu 2024 (China)	Knowledge acquisition	Scores of theoretical graduation examination (Max. S. not provided)	Cross sectional (N)	Dentistry	DUS/203/2	Traditional teaching (101)	Mean (SD) final scores: 71.94 (10.03)	Online virtual teaching (97)	Mean (SD) final scores = 72.23 (6.381)			—	
Gallardo 2022 (Spain)	Knowledge acquisition	Pre‐ and post‐intervention test scores. Max. S: 10	RCT (Y)	Paediatric dentistry	4th year DUS/86/2	Traditional lecture including video (37)	6.54 (2.54)	Flipped classroom (39)	7.77 (1.8)			Test group (flipped classroom)	0.006
Gerhardt‐Szep 2016 (Germany)	Knowledge acquisition	Number of correct answers in pre‐ and post‐intervention questionnaire. MQC. Max. S: 40	RCT (N)	Endodontics	DUS/101/2	Non facilitative tutor (51)	Mean = 22.2	Facilitative tutor (50)	Mean = 20.5			—	
	Attitude/Satisfaction/Perceptions	Questionnaire. 5‐point Likert scale					Mean (SD) (perceived support; tutor effectiveness; motivation) = 3.89 (0.43); 3.75 (0.49); 3.85 (0.46)		Mean (SD) (perceived support; tutor effectiveness; motivation) = 4.14 (0.45); 4.05 (0.50); 4.01 (0.66)			Test group (Facilitative tutor)	0.04–0.001
Godderidge 2019 (USA)	Knowledge acquisition	Pass rate in didactic exams	Cohorts comparison (N)	Prosthodontics	Last year DUS/381/2	Traditional instruction (141)	97.5%	Modified instruction (Student guided learning) (240)	99.5%			—	
González Cabezas 2015 (USA)	Knowledge acquisition	Scores in mid‐term and final exams. Max. S.: 100	Cohorts comparison (Y)	Cariology	1st year DUS/210/2	Instructor generated questions (106)	Mean (Midterm; final) = 82.4%; 86.3%	Student generated questions (104)	Mean (Midterm; final) = 86.6%; 89%			Test group (student generated questions) (only in midterm)	< 0.001
Guirado 2023 (Brazil)	Knowledge acquistion	Final test scores. 10 essay questions. Max. S.: 10	Cross over (Y)	Dental materials	2nd year DUS/88/2	Traditional lecture (88)	Mean (SD) = 6.29 (2.00)	Team‐based learning (88)	Mean (SD) = 6.23 (1.5)			—	
Haque 2021 (Pakistan)	Knowledge acquistion	Scores in Comprehensive final Test. MCQ. Max. S.: 100	RCT (Y)	Gross anatomy of head region	1st year DUS/49/2	Presentation with 2D images (24)	Mean (SD) = 31.41 (6.88)	Atlas with 3D rotating images (25)	Mean (SD) = 35.36 (6.02)			Test group (Atlas with 3D rotating images)	0.03
Hashemikamangar 2016 (Iran)	Knowledge acquistion	Pre‐ and post‐intervention test scores. Questions and case scenarios. Max. S.: 15	RCT (N)	Tooth discoloration	Senior Dental Students/63/2	(No activity specified) (31)	Detailed data (Mean and SD) not provided.	E‐learning (website with available material—case presentations, questions) (32)	Detailed data (Mean and SD) not provided.			Test group (E‐learning)	< 0.001
Hong 2023 (China)	Knowledge acquistion	Final course Scores. Max. S.: 30	Cohorts comparison (Y)	Oral medicine	3rd year DUS/180/2	Traditional offline teaching (86)	Mean = 22.39	Semi‐flipped classroom (94)	Mean = 25.33	Online live lectures (152)		Test group (Semi‐flipped Classroom)	< 0.001
	Attitude/Satisfaction/Perceptions	Ratings of teaching methods (offline/online/combination). Questionnaire. Max. S.: 10					Detailed data (Mean and SD) not provided.				Detailed data (Mean and SD) not provided.	Control group (offline method)	< 0.01
Howerton 2004 (USA)	Knowledge acquistion	Pre‐ and post‐intervention test scores. Max. S.: 20	RCT (Y)	Intraoral radiology	1st year DUS/75/3	Lecture with PowerPoint presentation only (24)	Post‐Test: Mean (SD) = 16.5 (2.414) Median = 17	Computer‐assisted instruction only (26)	Post‐Test: Mean (SD) = 17.000 (1.414) Median = 17	Computer‐assisted + Lecture (25)	Post‐Test: Mean (SD) = 16.880 (1.856) Median = 17	—	
	Attitude/Satisfaction/Perceptions	Questionnaire. 5‐point Likert scale										Test group (Computer‐assisted): Advantageous (92% Agree+Strongly agree); Preferred (54% Agree+Strongly agree)	< 0.001
Ilgüy 2014 (Turkey)	Knowledge acquistion	Final test scores (Assessment in 5 categories). Total = (A*5%)+ (B*15%)+ (C*20%)+ (D*25%)+ (E*35%). Max. S.: 100	Cohorts comparison (Y)	Oral diseases	4th year DUS/109/2	Lecture based learning (54)	Mean (SD) = 62.93 (29.85)	Case‐based learning (discussion sessions. No formal lectures) (55)	Mean (SD) = 72.74 (23.43)			Test group (Case‐Based) in categories D and E	0.014 0.026
Inamochi 2023 (Japan)	Knowledge acquistion	Individual and team test scores.MCQ (Max. S. not provided)	RCT (Y)	Prosthodontics	4th year DUS/195/3	Traditional on‐site Lecture (67)	Detailed data (Mean and SD) not provided.	On‐site flipped classroom (70)	Detailed data (Mean and SD) not provided.	Remote Flipped Classroom (58)	Detailed data (Mean and SD) not provided.	Test and Aditional groups (Onsite and Remote Flipped Classroom) (individual scores) Aditional group Remote FlippedClassroom) (team scores)	< 0.01 < 0.001
Iqbal 2024 (Pakistan)	Knowledge acquistion	Post‐intervention test scores. Max. S.: 90	Cohorts comparison (Y)	Operative dentistry (radiographic interpretation)	Final year DUS/82/2	Didactic lectures (39)	Mean (SD) = 44.4 (12.3)	Blended learning (orientation session + uploaded material + online group discussion + face‐to‐face clarification sessions) (37)	Mean (SD) = 61.0 (10.2)			Test group (blended learning)	< 0.001
Isherwood 2020 (UK)	Knowledge acquistion	Test questions covering the topic. Single best answer. Max. S.: 100%	RCT (Y)	Orthodontics	Final year DUS/61/2	Conventional didactic lecture (30)	Mean (SD) = 70.5% (8%)	Flipped classroom (access to videos + practical session) (31)	Mean (SD) = 72.8% (12.9%)			—	
Islam 2018 (Malaysia)	Knowledge acquistion	Post‐intervention test scores. True/false questions. Max. S.: 100%	Cohorts comparison (N)	Dental ergonomics	1st year DUS/50/2	Passive Learning (Lecture class) (25)	Mean = 89.58%	Active learning (Flipped classroom) (25)	Mean = 91.67%			—	
Jaffar 2023 (UAE)	Knowledge acquistion	Test Scores. MCQ (Max. S. not provided)	RCT (Y)	Head and neck anatomy	2nd year DUS/50/2	Standard lecture (25)	Median = 27	Interactive 3D sofware session (25)	Median = 31			—	
	Attitude/Satisfaction/Perceptions	Situational interest questionnaire every 20 min (5‐point scale)					Decrease in Situational interest after 40 min		Increase in Situational interest after 40 min				
Jeganathan 2020 (UK)	Knowledge acquistion	Improvement in learning comparing pre‐ and post‐intervention test scores. MCQ. Presented as %	RCT (Y)	Orthodontics	1st year DUS/70/2	Seminar‐based teaching without prior teaching (34)	Post‐test: Mean = 97.2%; Improvement: 20.6%	Seminar‐based teaching with blended approach (previous access to e‐learning resource) (34)	Post‐Test: Mean = 98.3%; Improvement: 19%			—	
	Attitude/perceptions	Questionnaire. 5‐point Likert scale					74% rated the teaching as Very Good		82% rated the teaching as Very Good				
Kalludi 2015 (India)	Knowledge acquistion	Post‐intervention test scores. Max. S.: 10	RCT (Y)	Physiology	1st year DUS/100/2	Didactic lecture followed by review from textbook (54)	Median (InterquartileRange) = 5.0 (3)	Didactic Lecture followed by video podcast session (46)	Median (InterquartileRange) = 6.0 (2)			Test Group (Video Podcast)	0.021
	Attitude/Satisfaction/Perceptions	Questionnaire. 5‐point Likert scale										Test group (Video podcast): might be useful (89% agreed)	
Karim 2022 (Pakistan)	Knowledge acquistion	Final semester test scores. (Max. S. not provided)	Cohorts comparison (Y)	Community dentistry (Subject I); Dental materials (Subject II)	2nd year DUS/84/2	Didactic lecture (43)	Mean (SD) (Subject I; Subject II) = 72.0 (8.08); 66.9 (9.08)	Interactive lecture (41)	Mean (SD) (Subject I; Subject II) = = 68.3 (9.04); 64.3 (10.04)			Test group (Interactive lecture) (only in Subject I)	0.04
Karimian 2024 (Iran)	Knowledge acquistion	Test scores in end of term exam. MCQ. Max. S.: 20	Cohorts comparison (Y)	Biochemistry	DUS and Medical students/160/2	Conventional face‐to face education. Lectures (Medical students) (95)	Mean (SD) = 13.65 (2.30)	Blended learning. Flipped classroom approach (Dental students) (49)	Mean (SD) = 13.77 (2.61)			—	
	Attitude/Satisfaction/Perceptions	Quality questionnaire. 6‐point Likert scale; Self‐evaluation questionnaire. 6‐point scale										Control group (Lectures) (effective content; questioning; active learning; feeling of the effectivenes) No difference in self‐evaluation scores	0.013–< 0.001
Kasabah 2016 (Saudi Arabia)	Knowledge acquistion	Test scores in pre‐ and post‐intervention and surprise objective test after 30 days. Max. S.: 25	RCT (N)	Cardio‐pulmonary resuscitation	DUS/40/2	Didactic lectures (20)	Mean (SD) (Post‐test; Surprise) = 15.2 (6.2); 10.9 (6.24)	Role‐play (20)	Mean (SD) (Post‐test; Surprise) = 17.8 (5.85); 16.2 (5.55)			Test group (role‐play) (only in surprise test)	< 0.05
Kavadella 2012 (Greece)	Knowledge acquistion	Pre‐ and post‐intervention test scores. Max. S.: 10	Controlled study (N)	Oral radiology	Final year DUS/47/2	Conventional face‐to‐face classroom lectures (22)	Post‐Test: Mean (SD) = 6.8636 (1.3903)	Blended learning with material uploaded to an educational online platform (24)	Post‐Test: Mean (SD) = 8.0875 (1.3820)			Test Group (Blended learning)	0.005
	Attitude/Satisfaction/Perceptions	Questionnaire. 5‐point Likert scale					Overall opinion on the course: Mean (SD) = 4.43 (0.662)		Overall opinion on the course: Mean (SD) = 4.46 (0.509)			—	
Kohli 2019 (Malaysia)	Knowledge acquistion	Test scores in short‐term (immediately after)and surprise long‐term test after 6 months. MCQ. (Max. S. not provided)	Cohorts comparison (Y)	Dental anatomy, dental hypersensitivity, dental caries, oral hygene	1st year DUS/60/3	Conventional lecture (20)	Mean (SD) (short‐term; Long‐term) = 163.70 (10.844); 147.50 (9.231)	Flipped classroom (20)	Mean (SD) (short‐term; Long‐term) = 153.25 (11.050); 145.05 (10.625)	Spaced learning (highly condensed learning content +breaks with distractor activities) (20)	Mean (SD) (short‐term; Long‐term) = 165.38 (13.677); 146.90 (10.319)	Aditional group (Spaced learning) (only short‐term)	0.003
	Attitude/perceptions	Questionnaire. 5‐point Likert scale					Mean (SD) (easy to remain concentrated; lecturer stopped discussion at right time; inspired to deal critacally; prectical relevance highlighted) = 3.05 (1.05); 3.45 (0.6); 3.3 (0.86); 4.1 (0.72)		Mean (SD) (easy to remain concentrated; lecturer stopped discussion at right time; inspired to deal critacally; prectical relevance highlighted) = 3.2 (0.89); 3.65 (0.75); 3.3 (0.8); 3.9 (0.79)		Mean (SD) (easy to remain concentrated; lecturer stopped discussion at right time; inspired to deal critacally; prectical relevance highlighted) = 3.6 (0.6); 3.6 (0.68); 3.6 (0.68); 3.55 (0.51)	Aditional group (Spaced learning) “easy to remain concentrated during the course” and “inspired to deal with the learning content critically”	0.01 0.04
Kunin 2014 ()	Attitude/Satisfaction/Perceptions	Pre‐ and post‐intervention perception questionnaire. 5‐point Likert scale	Cross over (Y)	Endodontics	1st‐year postgraduate dental students/169/2	Syncronous lectures (face‐to‐face and offsite) (169)	Data not clearly specified. Different sample size for each question Ability to learn: Mean (SD) = 3.5 (1,0) (n = 116) Level of comfort: Mean (SD) = 3.7 (0.98) (n = 123)	Asynchronous lectures (onsite and offsite) (169)	Data not clearly specified. Different sample size for each question Ability to learn: Mean (SD) = 3.9 (0.95) (n = 116) Level of comfort: Mean (SD) = 4.1 (0.90) (n = 123)			Test group (Asynchronous)	< 0.001
Lee 2018 (USA)	Knowledge acquisition	Pre‐ and post‐intervention test scores. MCQ. Max. S.: 6. Presented as percentage.	Cohorts comparison (Y)	Periodontal diagnosis and treatment planning	3rd year DUS/105/2	Conventional lecture (34)	Mean (SD) (Pre‐test; Post‐test) = 54%; 63%	Flipped classroom (69)	Mean (SD) (Pre‐test; Post‐test) = 64%; 78%			Test Group (Flipped Classroom)	< 0.01
Leinonen 2020 (Finland)	Knowledge acquisition	Scores in questionnaire at baseline and after 1st and 2nd activity. Max. S.: 100%	RCT (N)	Ergonomics	3rd year DUS/45/2	Lecture followed by video (23)	Mean (Beseline; 1st test; 2nd test) = 72%; 88%; 84%	Vidoeo followed by lecture (23)	Mean (Beseline; 1st test; 2nd test) = 72%; 82%; 84%			—	
Liao 2023 (Taiwan)	Knowledge acquisition	Score in final semester exam. Max. S.: 100	Cohorts comparison (Y)	Gross anatomy	3rd year DUS/70/2	Traditional lectures+Laboratory sessions (36)	Mean (SD) = 71.78 (12.68)	Asynchronous online videos+Laboratory smaller group sessions (34)	Mean (SD) = 82.21 (12.53)			Test group (Asynchronous online videos+Small group sessions)	0.0012
Liebermann 2022 (Germany)	Knowledge acquisition	2 Tooth recognition tests (Anterior and Posterior)	Cross over (Y)	Dental anatomy	1st semester DUS/82/2	Only conventional teaching in anterior teeth+additional virtual reality in posterior (Not provided)	Detailed data not provided.	Additional virtual reality glasses in anterior teeth+only conventional in posterior (Not provided)	Detailed data not provided.			—	
Liu 2020 (China)	Knowledge acquisition	Score in final exam. Max. S.: 100	RCT (Y)	Maxillary sinus floor augmentation	Postgraduate dental students/92/2	Traditional lecture (46)	Total score mean (SD) = 74.27 (3.07)	Small‐group case‐basedd and problem‐based learning (46)	Total Score Mean (SD) = 78.50 (3.21)			Test Group (Small‐group Case‐based and problem‐based learning)	< 0.01
	Attitude/Satisfaction/Perceptions	Questionnaire. Satisfaction rate expressed as percentage					Ratings: Mean (SD) = 76.1%; 73.9%; 87.0%; 60.9%; 39.1%; 82.6%; 52.2%; 73.9%; 32.6%		Ratings: Mean (SD) = 91.3%; 89.1%; 65.2%; 95.7%; 93.5%; 87.0%; 93.5%			Test group “makes learning more targeted and interesting”; “ehances ability to analyze and solve problems”; “helps improve clinical skills” “emphasizes on teamwork” Control group “decreases extracurricular workload”	< 0.001 0.0145
Llena 2018 (Spain)	Knowledge acquisition	Test scores in pre‐ and post‐intervention tests and surprise long‐term test after 6 months. Max. S.: 10	RCT (Y)	Cavity preparation	3rd year DUS/43/2	Traditional teaching (21)	Mean (SD) (Immediate post‐test; Long‐term) = 8.62 (1.59); 8.42 (1.50)	Augmented reality in addition to traditional teaching (20)	Mean (SD) (Immediate post‐test; Long‐term) = 8.65 (1.78); 8.75 (1.60)			—	
Luhrenberg 2022 (Germany)	Knowledge acquisition	Difference between Pre‐ and post‐intervention test scores. Max. S.: 1	RCT (Y)	Odontogenic tumors	1st, 2nd, 3rd and 5th DUS/71/2	E‐book (33)	Mean (SD) = ‐0.33 (3.11)	learning software (38)	Mean (SD) = ‐0.53 (3.11)			—	
Maggio 2012 (USA)	Knowledge acquisition	Final test scores and failure rate in final overall course grade Max. Score: 111. (Pass/fail threshold not provided)	RCT (Y)	Dental morphology	1st semenster DUS/120/2	Traditional classroom lectures + Advised access to a 3D Interactive Tooth Atlas (85)	Score: Mean (SD) = 88.1 (13.4); Failure rate: 5.8%	independent e‐learning interactive media module + Advised access to a 3D Interactive Tooth Atlas (35)	Score: Mean (SD) = 95.4 (10.4); Failure rate:0%			Test Group (Interactive e‐learning)	0.005
	Attitude/Satisfaction/Perceptions	Questionnaire. 5‐point Likert scale (and other types of questions)					Percieved value (believe the 3D Atlas was a helpful resource) (Strongly agree; Agree; Neutral; Disagree; Strongly Disagree): 22.4%; 41.6%; 21.2%; 8.2%; 7.0%		Percieved value (believe the 3D Atlas was a helpful resource) (Strongly agree; Agree; Neutral; Disagree; Strongly Disagree): 22.4%; 41.6%; 21.2%; 8.2%; 7.0%			Test group (Interactive additional material) recognized as a valuable learning resource by both groups (69.8% Test 63.3% Control)	
Mai 2022 (Korea)	Knowledge acquisition	Test scores in examination paper. Max. S.: 100	RCT (Y)	Principles of occlusal adjustment	2nd year DUS/60/2	Lecture with 2D Ilustrations (30)	Mean (SD) = 72.9 (10.0)	3D Simulation software (30)	Mean (SD) = 78.0 (11.0)			—	
Mai 2021 (Korea)	Knowledge acquisition	Test scores. Max. S.: 100	RCT (Y)	Guidance of the mandibular movement	2nd year DUS/60/3	Lecture only (20)	Mean (SD) = 82.7 (8.6)	Lecture + 3D Simulation (20)	Mean (SD) = 85.2 (7.4)	Simultaneous Lecture and 3D Simulation (20)	Mean (SD) = 90.7 (7.1)	Additional Group (Simultaneous Lecture and 3D Simulation)	< 0.001
Marei 2013 (Saudi Arabia)	Knowledge acquisition	Test Scores of theoretical and practical assessment. MCQ. Presented in terms of mean rank.	RCT (Y)	Local anesthesia	3rd year DUS/20/2	Traditional lecture (10)	Mean rank (Theoretical; Clinical) = 6.6; 8.85	Tutorial + practical demonstration + hands‐on practice on simulation phantom (10)	Mean rank (Theoretical; Clinical) = 14.4; 12.15			Test group (Simulation) (only in theoretical knowledge)	0.003
Markholm 2024 (Norway)	Knowledge acquisition	Scores in mid‐course and final tooth identification test. Presented in terms of number of Faults.	Cohorts comparison (N)	Dental anatomy	2nd year DUS/84/2	Traditional (hands‐on interaction with sets of extracted teeth) only (42)	Median (Q1‐Q2 (Mid‐course; final) = 12.0 (7.8–20.5); 1.0 (0.0–4.5)	Suplemental videos (aditional to hands‐on interaction) (42)	Median (Q1‐Q2) (Mid‐course; final) = 4.0 (0.0–8.0); 0.0 (0.0–2.5)			Test group ( Suplemental videos) (only in mid‐course)	< 0.001
Matos 2022 (Barzil)	Knowledge acquisition	Scores in pre‐ and post‐intervention practical cases tests	RCT (Y)	Paediatric dentistry—Dental tauma in primary dentition	3rd year DUS/36/3	Traditional lecture exclusively (12)	Post‐test: Mean (Diagnosis; Treatment) = 83.33%; 47.22%	educational mobile application exclusively (12)	Post‐test: Mean (Diagnosis; Treatment) = 93.06%; 51.39%	Traditional lecture + educational mobile application (12)	Post‐test: Mean (Diagnosis; Treatment) = 93.06%; 56.94%	—	
Meckfessel 2011 (Germany)	Knowledge acquisition	Failure rate in final test	Cohorts comparison (N)	Dental radiology	3rd year DUS/228/2	Traditional lecture (Cohorts 1 and 2) (Cohort1: 42; Cohort2: 48)	Failure rate (Cohort 1; Cohort 2) = 35.71%; 39.58%	E‐program (Cohorts 3 and 4) (Cohort3: 71; Cohort4: 67)	Failure rate (Cohort 3; Cohort 4) = 9.86%; 1.49%			Test Group (E‐program)	< 0.001
Mehta 2016 (UK)	Knowledge acquistion	Pre‐ and post‐intervention test scores	RCT (Y)	Orthodontic topics	4th year DUS/63/2	Not‐given electronic access to e‐learning material (31)	Mean (SD) (Pre‐test; Post‐test) = 6.39 (1.86); 6.64 (1.68)	Given electronic access to e‐learning material (32)	Mean (SD) (Pre‐test; Post‐test) = 6.42 (1.48); 6.71 (1.9)			—	
Mendoza Oropeza 2015 (Mexico)	Knowledge acquistion	Pre‐ and post‐intervention test scores	Randomized controlled trial (N)	Malocclusions of retained aAnd supernumerary teeth	4th year DUS/218/2	Traditional method (106)	Mean (SD) = 6.96 (1.26622)	Stereoscopic 3D imaging method. (112)	Mean (SD) = 7.82 (0.963963)			Test group (Stereoscopic 3D imaging)	0.00001
Mergany 2021 (Sudan)	Knowledge acquistion	Pre‐ and post‐intervention test scores	Cohorts comparison (Y)	Dental surgery forceps used for tooth extraction	8th and 9th 5th year DUS/67/2	No intervention (26)	Mean (Pre‐test; Post‐test) = 5.87; 5.42	Mobile application containing a gallery of the dental surgical forcep (31)	Mean (Pre‐test; Post‐test) = 5.94; 8.34			Test group (Mobile application)	< 0.001
Messina 2022 (USA)	Knowledge acquistion	Scores in midterm examination. Max. S.: 50	Cohorts comparison (Y)	Cariology	1st year DUS/240/2	Live, in‐person lecture format (120)	Mean (SD) = 42.636 (3.64)	Combination of synchronous online lectures and asynchronous interactive presentations in education platform Nearpod (120)	Mean (SD) = 44.933 (2.626)			Test group (Nearpod interactive education platform)	< 0.001
Miller 2013 (USA)	Knowledge acquistion	Unit exam and final exam scores. MCQ. (Max. S. not provided)	Cohorts comparison (Y)	Physiology course	5th year DUS/120/2	Traditional didactic lecture (120)	Mean (SD) (Unit; Final) = 78.66 (5.58); 70.58 (13.06)	Engaging lecture methods (120)	Mean (SD) (Unit; Final) = 87.25 (2.18); 93.49 (4.55)			Test group (Engaging lectures)	< 0.05
	Attitude/Satisfaction/Perceptions	Questionnaire. 5‐point Likert scale					Detailed data not provided		Detailed data not provided			Test group (Engaging lectures)	< 0.0001
Miller 2015 (USA)	Knowledge acquistion	Unit exam and final exam scores. Presented as percentage of correct answers	Cohorts comparison (Y)	Dental physiology course	5th year DUS/120/2	No video class (120)	Mean (basic science;clinical application) = 91%; 79%	Clinical scenario video modules (120)	Mean (basic science;clinical application) = 91.5%; 88.1%			Test group (Video modules) (only in clinically related questions)	< 0.05
	Attitude/Satisfaction/Perceptions	Questionnaire. 5‐point Likert scale					Detailed data not provided		Detailed data not provided			Test group (Video modules)	< 0.05
Mirzaei 2024 (Iran)	Knowledge acquistion	Test scores in post‐intervention and retention (after 6 months) tests and self‐reported performance. Max. S.: 46	RCT (Y)	Infection control training course	5th year DUS/70/2	Traditional lecture (35)	Knowledge:Mean (SD) (Post‐test; Retention) = 26.48 (4.63); 24.52 (3.96) Self‐reported performance: Mean (SD) (Post‐test; Retention test) = 22.08 (1.85); 17.8 (1.57)	Concept map (35)	Knowledge: Mean (SD) (Post‐test; Retention) = 30.29 (8.77); 27.54 (7.39) Self‐reported performance: Mean (SD) (Post‐test; Retention test) = 22.97 (1.25); 19.29 (1.13)			Test group (Concept map) in knowldge and in self‐reported performance	0.022 < 0.001
Moazami 2014 (Iran)	Knowledge acquistion	Test scores in post‐intervention tests and retention test after 2 months. MCQ and essay questions. Max. S.: 30	RCT (Y)	Rotary instrumentation of root canals	5th year DUS/35/2	Traditional learning (20)	Mean (SD) (Post‐test; Retention) = 19.25 (5.19); 17.26 (3.35)	Virtual learning (15)	Mean (SD) (Post‐test; Retention) = 22.45 (4.41); 19.65 (4.88)			—	
Murthykumar 2015 (India)	Knowledge acquistion	Test scores. (Max. S. not provided)	Cohorts comparison (Y)	Biostatistics	Postgraduate Students/88/2	Traditional learning (44)	Mean (SD) = 53.48 (8.382)	Video based learning (44)	Mean (SD) = 66.60 (8.920)			Test group (Video based learning)	< 0.001
Naik 2022 (India)	Knowledge acquistion	Scores in pre‐ and post‐intervention test scores MCQ. Max. S.: 10 and written case‐based examination Presented in terms of mean rank.	Controlled trial (Y)	Oral medicine and radiology “Periapical inflammatory diseases”	3rd year DUS/40/2	Traditional learning (20)	Mean (SD) (Pre‐test; Post‐test) = 4.6 (1.5); 5.8 (1.2) Difference Pre‐Post = ‐25.30% Written test: Mean rank = 11.2	Flipped classroom (20)	Mean (SD) (Pre‐test; Post‐test) = 5.1 (1.5); 8.1 (1.3) Written test: Mean rank = 29.8 Difference Pre‐Post = ‐57.73%			Test group (Flipped classroom)	0.0001
Narasimhan 2023 (UAE)	Knowledge acquistion	Scores in theory and oral histopathology slide test. (Max. S. not provided)	Cohorts comparison (N)	Preclinical lab course	1st/2nd year DUS/251/2	Traditional methods of lab teaching (131)	Detailed data not provided.	Online methods (120)	Detailed data not provided.			Test group (Online methods)	< 0.001
Nikkerdar 2023 (Iran)	Knowledge acquistion	Test scores immediately and two months after intervention.Max. S.: 10	RCT (Y)	Radiographic differential diagnosis of maxillofacial lesions	DUS/50/2	Lecture‐based instruction (25)	Mean (SD) (Immediate; 2 Months) = 5.26 (0.86); 5.16 (0.40)	Smartphone application (25)	Mean (SD) (Immediate; 2 Months) = 7.54 (0.45); 7.10 (0.21)			Test group (Smartphone application)	< 0.001
Nilsson 2011 (Sweden)	Knowledge acquistion	Test scores immediately and eight months after training. (Max. S. not provided)	RCT (Y)	Oral radiology	7th/9th semester DUS/45/2	Traditional teaching (25)	Mean (SD) = (Immediate; 8 Months) = 3.80 (1.35); 3.92 (1.32)	Simulator‐based training program (for interpretation of spatial relations in radiographs) (20)	Mean (SD) = (Immediate; 8 Months) = 4.15 (1.14); 4.00 (0.86)			—	
Nirmal 2020 (India)	Knowledge acquistion	Test scores at the end of every module. MCQ. Max. S.: 10	RCT (Y)	Non‐pharmacological behaviour management (Module I); Fluorides (Module II)	Final year DUS/60/2	Didactic lectures (30)	Module I: Mean (Session 2; Session 4) = 2.9; 6.6 Module II: Mean (Session 2; Session 4) = 4.6; 5.1	Didactic lectures + Puzzle activities (30?)	Module I: Mean (Session 2; Session 4) = 4.2; 6.1 Module II: Mean (Session 2; Session 4) = 9.3; 6.0			Test group (Puzzle activities) (except Session 4 in Module I)	0.032–< 0.001
Nkenke 2012 (Germany)	Knowledge acquistion	Test scores. MCQ. Max. S.: 20	RCT (Y)	Theoretical radiological science	3rd year DUS/42/2	Traditional face‐to‐face course (21)	Mean (SD) = 18.6 (1.2)	Technology enhanced learning (21)	Mean (SD) = 18.3 (1.3)			—	
	Attitude/Satisfaction/Perceptions	Questionnaire. 6‐point Likert scale					Confident in being successful in the exam: Mean (SD) = 4.5 (1.4)		Confident in being successful in the exam: Mean (SD) = 3.3 (1.7)			Control group (face ‐to‐face) in confidence in being successful in the exam Students of both groups rated e‐learning positively. Still, considered face‐to‐face lectures the basis of education at university	0.020
Oh 2022 (USA)	Knowledge acquistion	Scores and pass rates in practical examination. Max. S.: 80 (Pass/fail threshold not provided)	Cohorts comparison (Y)	Periodontal instrumentation	2nd year DUS/389/2	Traditional on‐site simulation‐based learning (Two cohorts ) (133 (2022 class))	Scores: Mean = 64.66 Pass rate: 72%	Remote Simulation‐based learning (126)	Scores: Mean = 67.98 Pass rate: 84%			Test group (Remote)	< 0.05
Oh 2023 (USA)	Knowledge acquistion	Final examination scores. MCQ and case‐based questions. Max. S.: 200	Cohorts comparison (Y)	Periodontics	2nd year DUS/259/2	Classroom lecture (133)	Mean (SD) = 160.8 (20.4)	Online lecture (126)	Mean = 166.4 (17.4)			Test group (Online)	0.019
Paul 2019 (Malaysia)	Knowledge acquistion	Number of correct answers in surprise test one week after. OSCE type questions Max. S: 20	Controlled study (Y)	Odontogenic infections	5th year DUS/45/2	Traditional lecture (23)	Mean (SD) = 5.21 (1.20)	Blended Classroom (22)	Mean (SD) = 9.50 (0.51)			Test group (Blended classroom)	< 0.001
Pérez‐Higueras 2023 (Spain)	Knowledge acquistion	Scores in end of term exam 2 months after. MCQ. Max. S.: 10	Cohorts comparison (Y)	Root canal morphology classification system	3rd year DUS/88/2	Traditional lecture (88)	Mean (SD) = 6.9 (2.3)	Small‐group practical seminar (88)	Mean (SD) = 7.7 ( 2.5)			Test group (Small‐group practical seminar)	0.006
Peroz 2009 (Germany)	Knowledge acquistion	Test scores immediately and six weeks after. MCQ	RCT (N)	Instrumental occlusal analysis	1st/3rd semester DUS/85/2	Lecture group (37)	Detailed data (Mean and SD) not provided.	Computer assisted self‐learning (48)	Detailed data (Mean and SD) not provided.			Control group (Lecture) (only in immediate test)	0.011
	Attitude/Satisfaction/Perceptions	Scoring questionnaire. Max. S.: 10					Mean (Enjoyed; Clearly structured; Suitable content) = 8.8; 9; 8.6		Mean (Enjoyed; Clearly structured; Suitable content) = 7.1; 7.9; 7.1			Control group (Lecture)	< 0.001
Priyam 2020 (India)	Knowledge acquistion	Pre‐ and post‐intervention test scores	RCT (Y)	Dentition status and treatment need	3rd year DUS/38/2	Routine teaching group (17)	Mean rank = 10.47	Fish bowl and one‑minute preceptor (21)	Mean rank = 26.81			Test group (Fish bowl and one‑minute preceptor)	< 0.001
Puranik 2023 (USA)	Knowledge acquistion	Scores in OSCE. Max. S.: 10	Cohorts comparison (Y)	Traumatic dental injuries	Predoctoral students and advanced standing students/240/2	Traditional (121)	Mean (SD) (Traumatic Dental Injuries; Pulp therapy; Non‐pharmacologic behavior management = 7.7 (1.5); 7.6 (1.5); 8.7 (1.1)	Problem‐based learning (119)	Mean (SD) (Traumatic Dental Injuries; Pulp therapy; Non‐pharmacologic behavior management = 8.2 (1.2); 8.5 (1.3); 9.1 (0.9)			Test group (Problem‐based learning)	0.007–< 0.001
	Attitude/Satisfaction/Perceptions	Self‐perceived learning questionnaire. 5‐point Likert scale				Traditional (84)	Students with positive perceptions (Guidance; Diagnostic skills; Radiographic assessment; Overall experience) = 90%; 91%; 88%; 95%	Problem‐based learning (82)	Students with positive perceptions (Guidance; Diagnostic skills; Radiographic assessment; Overall experience) = 95%; 93%; 93%; 92%			Test group (Problem‐based learning) in faculty guidance, improved diagnostic skills and radiographic assessment	< 0.05
Qazi 2019 (Pakistan)	Knowledge acquistion	Asssessment scores. Theory, practical (Max. S.: 100) and total (Max. S.: 200) Pass rate. Theory, practica (threshold: 50) and total	Cohorts comparison (Y)	Science of dental materials	1st year DUS/98/2	Traditional teaching in classroom (47)	Score: Mean (SD) (Theory; Practical; Total) = 65.26 (13.18); 65.13 (12.70); 130.02 (24.22) Pass rate (Theory; Practical; Total) = 87.23%; 85.11%; 80,85%	Modified teaching approach: Rotational placement on clinics (51)	Score: Mean (SD) (Theory; Practical; Total) = 61.80 (11.47); 67.49 (8.17); 129.29 (18.62) Pass rate (Theory; Practical; Total) = 90.20%; 100%; 90.20%			—	
	Attitude/Satisfaction/Perceptions	Focus group meetings										Test group (Clinical rotations). (Satisfaction levels; Positive perceptions; Enhanced understanding; Provided context)	
Ramseier 2012 (Switzerland)	Knowledge acquistion	Scores in pre‐ and post‐intervention tests and retention test after 5 months. Presented as percentage of correctly answered questions	Controlled Trial (N)	Basic principles of fixed prosthodontics	3rd year DUS/66/2	Study using conventional paper manuscript (17–2003 cohort, 16–2006 cohort)	2003: Mean (SD) (Post‐test; Retention) = 69.0 (4.9); 53.4 (9.7) 2006: Mean (SD) (Post‐test; Retention) = 55.2 (10.7); 51.7 (7.3)	Study using web‐based application (17–2003 cohort, 16–2006 cohort)	2003: Mean (SD) (Post‐test; Retention) = 69.0 (7.8); 48.3 (7.5) 2006: Mean (SD) (Post‐test; Retention) = 51.7 (13.2); 48.3 (4.0)			—	
Rekha 2017 (India)	Knowledge acquistion	Test scores. MCQ. Max. S.: 10	RCT (Y)	Public health dentistry	Final year DUS/38/2	Lecture (19)	Mean (SD) = 4.84 (1.17)	Problem‐based learning (19)	Mean (SD) = 6.63 (1.80)			Test group (Problem‐based learning)	0.001
Robson 2015 (UK)	Knowledge acquistion	Test scores. MCQ. Max. S.: 20	RCT (Y)	Orthodontics	2nd year DUS/74/2	Traditional didactic lecture (37)	Mean (SD) = 2.8 (2.0)	Integrated audience response system during lecture (37)	Mean (SD) = 3.6 (2.3)			Test group (Integrated audience response system)	< 0.001
Sagsoz 2017 (Turkey)	Knowledge acquistion	Scores in pre‐ and post‐intervention tests and retention test after 3 weeks. Max. S.: 100	RCT (N)	Adhesion and bonding agents	3rd year DUS/50/2	Lecture‐based learning (25)	Mean (SD) (Post‐test; Retention) = 54.60 (10.30); 51.20 (8.91)	Jigsaw learning (25)	Mean (SD) (Post‐test; Retention) = 57.40 (6.63); 57.80 (8.91)			Test group (Jigsaw) (only in retention test)	0.012
Salian 2022 (India)	Knowledge acquistion	Test scores. MCQ. (Max. S. not provided)	Controlled trial (Y)	Oral pathology	3rd year DUS/90/2	Traditional (45)	Mean (SD) = 5.318692 (1.478917)	Live‐field teaching (45)	Mean (SD) = 5.080374 ( 1.98173)			Control group (Traditional)	0.032
Santhosh 2024 (India)	Knowledge acquistion	Scores in pre‐ and post‐intervention tests and two retention tests after 1 and 3 months. MCQ. Max. S.: 20	RCT (Y)	Principles of health education	Final year DUS/90/2	Lecture‐based learning (45)	Mean (SD) (Post‐test; 1month retention; 3month retention) = 17.84 (1.40); 11.04 (1.86); 8.02 (1.03)	Spaced repetition learning with mobile flashcard application (45)	Mean (SD) (Post‐test; 1month retention; 3month retention) = 17.71 (1.53); 14.58 (2.75); 11.07 ( 0.89)			Test group (Spaced learning with mobile application) (only in retention tests)	≤ 0.001
	Attitude/Satisfaction/Perceptions	Questionnaire. 5‐point Likert scale					Cumulative mean perception score (SD) = 25.07 (1.36)		Cumulative mean perception score (SD) = 38.40 (9.43)			Test group (Spaced learning with mobile application)	0.001
Schönwetter 2016 (Canada)	Knowledge acquistion	Pre‐ and post‐intervention test scores	RCT (Y)	Root canal obturation	2nd year DUS/28/2	Traditional live lecture (14)	Mean (SD) = (Recall; Recognition) 5.00 (2.18); 2.79 (0.70)	Voice‐over‐screen‐captured lecture delivered online (14)	Mean (SD) = (Recall; Recognition) 4.64 (1.15); 2.57 (1.28)			—	
	Attitude/Satisfaction/Perceptions	Questionnaire. 5‐point Likert scale					Mean (SD) (Satisfaction; Self‐reported extent of attendance; self‐reported engagement) = 3.64 (0.67); 2.79 (0.70); 3.55 (0.69)		Mean (SD) (Satisfaction; Self‐reported extent of attendance; self‐reported engagement) = 3.15 (1.35); 3.64 (0.67); 3.62 (0.87)			—	
Shetty 2023 (UAE)	Knowledge acquistion	Scores in online test one week after intrvention. MCQ. Max. S.: 15	RCT (Y)	Fully guided implant planning	Final year DUS/90/3	Didactic lectures only (30)	Overall scores: Mean (SD) = 10.57 (1.68)	Hands‐on session of virtual implant planning in addition to lectures and video (30)	Overall scores: Mean (SD) = 13.57 (1.22)	Video in addition to the didactic lectures (30)	Overall scores: Mean (SD) = 10,33 (2,26)	Test group (Hands‐on session of Vrtual implant planning)	0.01
	Attitude/Satisfaction/Perceptions	Questionnaire. 3‐point Likert scale										Test group (Virtual implant planning): easier to familiarize with process (94.44%); easier to get understanding of technical points and difficulties (90%)	
Shigli 2017 (India)	Knowledge acquistion	Scores in OSPE	RCT (Y)	Prosthodontics related to impression making	2nd year DUS/73/2	Lecture only (32)	Mean (SD) = 13.97 (3.64)	Early clinical exposure (lectures accompanied by video demonstration of clinical procedure) (41)	Mean (SD) = 14.69 (3.32)			—	
Shiota 2021 (Japan)	Knowledge acquistion	Scores in written test. Max. S.: 158	RCT (Y)	Sports dentistry	5th year DUS/166 (Tokyo) 139 (Saitama)/2	Conventional video lecture (63)	Mean (2017; 2018;2019) = 47.0; 52.5; 46.5	Computer‐assisted learning (67)	Mean (2017; 2018;2019) = 91.0; 84.0; 86.5			Test group (Computer‐assisted learning)	< 0.001
	Attitude/Satisfaction/Perceptions	Questionnaire. 4‐point Likert scale										—	
Shqaidef 2021 (Jordan)	Knowledge acquistion	Test scores. Max. S.: 10	RCT (Y)	Treatment planning in orthodontics	4th year DUS/94/3	Live lecture (32)	Mean (SD) = 7.7 (1.56)	Video recorded lecture (33)	Mean (SD) = 8.0 (1.75)	Audio recorded lecture and printed copy of lecture slides (29)	Mean (SD) = 6.8 (1.73)	Control and Test groups (Live and Video recorded lecture)	< 0.05
	Attitude/Satisfaction/Perceptions	Questionnaire. 5‐point Likert scale										—	
Signori 2019 (Brazil)	Knowledge acquistion	Scores in post‐intervention test (Max. S.: 5) and percentage of correct answers in retention test 6 months after	RCT (Y)	Diagnosis and management of tooth restorations	3rd year DUS/40/2	Lecture (20)	Post‐intervention: 3.75 (1.29) Retention: Mean = 59.2%	Diagnostic workshop after lecture (20)	Post‐intervention: 3.95 (1.00) Retention: Mean = 71.9%			Test group ( Diagnostic workshop) (only in retention test)	0.027
	Attitude/Satisfaction/Perceptions	Self perception questionnaire.4‐point Likert scale					Detailed data not provided		Detailed data not provided.			—	
Slaven 2019 (USA)	Knowledge acquistion	Improvement as difference between pre‐ and post‐intervention test scores	RCT (Y)	Behavior guidance techniques in pediatric dentistry	2nd year DUS/96/3	Traditional instruction with lecture only (32)	Mean improvement = 4.03	Flipped classroom and video (32)	Mean improvement = 4.00	Traditional instruction with videos (32)	Mean improvement = 3.61	—	
	Attitude/Satisfaction/Perceptions	Rating questionnaire. 4‐point Likert scale					Mean (Course satisfaction; Module usefulness) = 3.19; 3.19		Mean (Course satisfaction; Module usefulness) = 3.25; 3.31		Mean (Course satisfaction; Module usefulness) = 3.03; 3.06	—	
Soltanimehr 2019 (Iran)	Knowledge acquistion	Scores in theoretical post‐intervention test (MCQ) and immediate and retention OSCE after 2 months. (Max. S. not provided)	RCT (Y)	Radiographic interpretation of bony lesions of the jaw	4th year DUS/39/2	Traditional lecture‐based education (19)	Theoretical: Mean (SD) (Immediate; 2month) = 14.89 (0.99); 14.45 (0.83) OSCE: Mean (SD) (Immediate; 2month) = 14.71 (0.92); 14.18 (0.95)	Virtual teaching (20)	Theoretical: Mean (SD) (Immediate; 2month) = 16.60 (0.91); 15.88 (0.78) OSCE: Mean (SD) (Immediate; 2month) = 15.13 (0.78); 14.75 (0.87)			Test group (Virtual teaching) (only in theoretical tests)	< 0.001
Stamm 2019 (Germany)	Knowledge acquistion	Scores in post‐intervention test and NDE	Cohorts comparison (Y)	Orthodontics	7th/8th semester DUS/117/2	No tablet (64)	Detailed data not provided	Use of tablet PC (53)	Detailed data not provided			Test group (Use of tablet PC)	0.002
Susarla 2004 (USA)	Knowledge acquistion	Percentage of students publishing an abstract in Journal of Dental Research	Cohorts comparison (N)	Not specified	DUS/Not specified/2	Non‐problem‐based learning group (Not specified)	Mean = 40.2%	Problem‐based learning (Not specified)	Mean = 28.7%			—	
Tan 2009 (UK)	Knowledge acquistion	End‐of‐year examination grades. (Max. S. not provided)	Cohorts comparison (N)	Radiology	1st year DUS/140/3	Face‐to‐face lectures (5)	Mean (SD) = 2.57 (0.98)	E‐learning modules (95)	Mean (SD) = 1.81 (1.01)	Both face‐to‐face and e‐leaning. (40)	Mean (SD) = 1.51 (0.98)	Aditional group (Both e‐learning and face‐to‐face)	< 0.01
	Attitude/Satisfaction/Perceptions	Rating questionnaire. 4‐point scale					Ratings (Excellent; Good; Adequate; Unsatisfactory) = 62%; 33%; 5%; 0%		Ratings (Excellent; Good; Adequate; Unsatisfactory) = 42%; 40%; 15%; 3%			—	
Thangavelu 2021 (India)	Knowledge acquistion	Final examination scores. Max. S.: 200	Cohorts comparison (N)	Pharmacology	2nd year DUS/278/2	Conventional Teaching (92)	Mean (SD) = 147.7 (1.228)	Flipped Classroom (2 cohorts) (92 (2016) 94 (2017))	Mean (SD) (2016; 2017) = 147.6 (1.28); 154.95 (1.015)			—	
Thurzo 2010 (Slovakia)	Knowledge acquistion	Test scores of one immediate and two retention examinations after 12 and 24 months (Max. S. not provided)	RCT (N)	Cephalometric analysis in orthodontics	4th year DUS/24/2	Manual Learning (12)	Mean = 6.0833	E‐learning (12)	Mean = 8.2500			Test group (E‐learning)	0.00003
Tuil 2023 (France)	Knowledge acquistion	Post‐intervention test scores. MCQ. Max. S.: 15	RCT (Y)	Oral rehabilitation of edentulous patients	3rd year DUS/89/2	Conventional lectures and practical exercises (58)	Mean (SD) = 7.4 (2.3)	Addition of game‐based training (31)	Mean (SD) = 9.2 (2.1)			Test group (game‐based training)	0.0004
Varughese 2024 (India)	Knowledge acquistion	Post‐intervention test scores. MCQ. Max S.: 10	RCT (Y)	Cement manipulation	1st year DUS/57/3	Traditional lecture (19)	Mean (SD) = 6.30 (1.23)	Smart class (delivered with audiovisual aids) (19)	Mean (SD) = 6.35 (0.95)	Flipped learning (19)	Mean (SD) = 8.11 (1.08)	Aditional group (Flipped learning)	< 0.001
Vollath 2020 (Germany)	Knowledge acquistion	Scores in post‐intervention and retention test after 6 months. OSCE. Max. S.: 100	Cohorts comparison (Y)	Smoking cessation	DUS/55/2	Standard teaching (27)	Mean (SD) (Post‐test; Retention test) = 41.8 (1.5); 36.5 (1.4)	New protocol: Podcast, interactive lecture, seminar, and small‐group sessions with role‐play interactions (28)	Mean (SD) (Post‐test; Retention test) = 67.1 (1.9); 52.7 (1.8)			Test group (New protocol)	< 0.001
Wang 2019 (China)	Attitude/Satisfaction/Perceptions	Rating questionnaire. 4‐point scale	Cohorts comparison (Y)	Medical physiology	2nd year DUS/156/2	Traditional (58)	Mean (SD) = 3.1 (0.5)	Inverted classroom (65)	Mean (SD) = 3.7 (1)			Test group (Inverted classroom)	< 0.05
Wang 2021 (Japan)	Attitude/Satisfaction/Perceptions	Individual and Team readiness assurance tests. Max. S.: 100	RCT (Y)	Removable prosthodontics	4th year DUS/137/2	Lecture group (67)	Mean (SD) = 36.1 (15.8)	Flipped classroom (70)	Mean (SD) = 46.1 (18.3)			Test group (Flipped classroom)	0.001
Wimalarathna 2021 (Sri Lanka)	Knowledge acquistion	Post‐intervention test scores. MCQ and OSCE. Max. S.: 100	RCT (Y)	Traumatic dental injuries	Final year DUS/45/2	Lecture group (23)	Mean (SD) = 62.8 (8.6)	Interactive Internet tool. Dental Trauma Guide (21)	Mean (SD) = 66.3 (15.1)			Test group (Dental Trauma Guide)	0.005
Wu 2022 (China)	Knowledge acquistion	Post‐intervention test scores. (Max. S. not provided)	RCT (Y)	Oral medicine “Erythema multiforme”	4th year DUS/60/2	Traditional lecture (30)	Detailed data not provided	Online learning combined with case‐based discussion (30)	Detailed data not provided			Test group (Online case‐based discussion)	< 0.05
Xiao 2018 (USA)	Knowledge acquistion	Test scores. MCQ. Presented as percentage	Cohorts comparison (Y)	Physiology	1st year DUS/283/2	Traditional Class (142)	Mean = 69%	Flipped classroom (141)	Mean = 80%			Test group (Flipped classroom)	< 0.01
Yusifli 2024 (Turkey)	Attitude/Satisfaction/Perceptions	Questionnaire. 5‐point Likert scale	RCT (Y)	Vazirani–Akinosi technique of inferior alveolar nerve block	Final year DUS/91/2	No further training (42)	Total score: Mean (SD) = 40.33 (4.29)	Practical training (49)	Total score: Mean (SD) = 42.47 (2.99)			Test group (Practical training)	0.006
Zhai 2022 (China)	Knowledge acquistion	Scores in final theory test. Max. S.: 10	Cohorts comparison (Y)	Oral pathology	1st and 2nd year DUS/160/2	Traditional lecture‐based teaching (72)	Mean (SD) = 5.196 (0.2170)	Presentation–Assimilation–Discussion class (88)	Mean (SD) = 7.978 ( 0.1349)			Test group (Presentation—Assimilation—Discussion)	< 0.05
	Attitude/Satisfaction/Perceptions	Questionnaire. 5‐point Likert scale				Traditional lecture‐based teaching (68)		Presentation–Assimilation–Discussion class (79)				Test group (Presentation—Assimilation—Discussion) in Stimulated learning interest, Improved self‐learning ability, Improved communication abilities, Improved ability to comprehensively use knowledg,e Improved critical thinking, Improved problem analysis and solving ability, Improved innovation,Promoted reflection, Realize learning goals	0.003–< 0.001
Zhang 2024 (China)	Knowledge acquistion	Scores in theoretical pre‐ and post‐intervention tests and retention test after 3 months	RCT (Y)	Pathology and radiology	2nd and 3rd DUS/63/3	Traditional (21)	Mean (SD) (Post‐test; Retention) = 72.57 (3.84); 65.29 (4.62)	KoPa WiFi EDU system. (21)	Mean (SD) (Post‐test; Retention) = 80.43 (3.41); 70.10 (5.02)	KoPa WiFi EDU system and a CBCT software (21)	Mean (SD) (Post‐test; Retention) = 89.29 (4.55); 77.95 (4.83)	Aditional group (KoPa WiFi and CBCT software) (in post‐test) Test and Aditional groups (KoPa WiFi, KoPa WiFi+CBCTsoftware) (in retention test)	< 0.001 < 0.05
	Attitude/Satisfaction/Perceptions	Questionnaire. 5‐point Likert scale					Final satisfaction survey for learning effects: Mean (SD) = 4.13 (0.64)		Final satisfaction survey for learning effects: Mean (SD) = 4.53 (0.52)		Final satisfaction survey for learning effects: 4.80 (0.41)	Test and Aditional groups (KoPa WiFi, KoPa WiFi+CBCTsoftware)	< 0.05
Zhong 2023 (China)	Knowledge acquistion	Post‐intervention test scores. Max. S.: 100	Cohorts comparison (Y)	Oral histopathology course	1st year DUS/214/2	Traditional classroom (104)	Mean (SD) = 76.7 (10.93)	Flipped classroom (110)	Mean (SD) = 83.79 (11)			Test group (Flipped classroom)	< 0.0001
	Attitude/Satisfaction/Perceptions	Questionnaire. 5‐point Likert scale					Mean (SD) = 4.423 (0.01366)		Mean (SD) = 4.599 (0.1027)			Test group (Flipped classroom)	< 0.01
Zhong 2021 (China)	Knowledge acquistion	Post‐intervention test scores. Max. S.: 100	Cohorts comparison (Y)	Oral histopathology	3rd, 4th and 5th DUS/192/2	Traditional (face‐to‐face) (98)	Mean (SD) = 77.25 (7.55)	E‐Learning platform and virtual simulation experiment teaching (94)	Mean (SD) = 82.94 (10.76)			Test group (Remote learning and virtual technology)	< 0.01

Abbreviations: DUS, dental undergraduate students; Max. S, maximum score; MCQ, multiple choice questions; NDE, National Dental Examination; OSCE, objective structured clinical examination; OSPE, objective structured practical examination.

**TABLE 2:** Characteristics of studies included in the meta‐analysis by subgroups.

Subgroup	Study	Country	Time for exam	Academic year	Maximum test score	Control group (Traditional lecture)					Experimental group				
						Final n	Mean score	SD	Normalised mean	Normalised SD	Final n	Mean score	SD	Normalised mean	Normalised SD
1. Asynchronous independent learning	Howerton 2004	USA	2 weeks after completion	1	20	24	16.5	2.4	82.5	12.0	26	17	1.4	85.0	7.0
	Liao 2023	Taiwan	Completion of course	3	100	36	71.78	12.68	71.8	12.7	34	82.21	12.53	82.2	12.5
	Maggio 2012	USA	Completion of module	1	111	85	88.1	13.4	79.4	12.1	35	95.4	10.4	85.9	9.4
	Moazami 2014	Germany	2 months after completion	5	30	20	17.26	3.35	57.53	11.17	15	19.65	4.88	65.5	16.27
	Nkenke 2012	Germany	2 weeks after completion	3	20	21	18.6	1.2	93.0	6.0	21	18.3	1.3	91.5	6.5
2. Synchronous blended learning	Deepak Nallaswamy 2019	India	Completion of course	Final year	200	75	130.93	9.12	65.5	4.6	75	150.35	10.93	75.18	5.5
	Iqbal 2024	Pakistan	—	Final year	90	39	44.4	12.3	49.3	13.7	37	61	10.2	67.78	11.3
	Isherwood 2020	UK	—	Final year	100	30	70.5	8	70.5	8.0	31	72.8	12.9	72.80	12.9
	Kavadella 2012	Greece	Completion of course	Final year	10	22	6.86	1.4	68.6	14.0	24	8.1	1.4	81.0	14.0
	Naik 2022	India	Completion of module	3	10	20	5.82	1.2	58.2	12.0	20	8.1	1.3	81.0	13.0
	Paul 2019	Malaysia	1 week after completion	5	10	23	5.21	1.2	52.1	12.0	22	9.5	0.5	95.0	5.0
	Varughese 2024	India	—	1	10	19	6.3	1.23	63.0	12.3	19	8.11	1.08	81.10	10.8
	Zhong 2023	China	End of semester	3	100	104	76.7	10.93	76.7	10.9	110	83.8	11	83.80	11.0
3. Synchronous small‐group learning	Arias 2016	USA	Completion of the course	1	5	68	4.3	1.2	86.0	24.0	66	4.49	1.11	89.80	22.2
	Echeto 2015	USA	5 semesters after completion	Senior	1	79	0.7	0.09	70.0	9.0	78	0.76	0.08	76.0	8.0
	Guirado 2023	Brazil	—	2	10	88	6.29	2	62.9	20.0	88	6.23	1.5	62.30	15.0
	Ilgüy 2014	Turkey	Completion of course	4	100	54	62.93	29.85	62.93	29.85	55	72.74	23.43	72.74	23.43
	Liu 2020	China	3 months after completion	Postgraduate	100	46	74.27	3.07	74.3	3.1	46	78.5	3.21	78.50	3.2
	Rekha 2017	India	End of session	Final year	10	19	4.84	1.17	48.4	11.7	19	6.63	1.8	66.30	18.0
	Sagsoz 2017	Turkey	3 weeks after completion	3	100	25	51.2	8.91	51.2	8.91	25	57.80	7.91	57.80	7.91
	Messina 2022	USA	Midterm examination	1	50	120	42.6	3.64	85.2	7.3	120	44.93	2.62	89.86	5.2
	Kasabah 2016	Saudi Arabia	30 days after completion	—	25	20	10.9	6.2	43.6	24.8	20	16.2	5.6	64.80	22.4
	Mirzaei 2024	Iran	6 months after completion	5	46	35	24.52	3.96	53.3	8.6	35	27.54	7.4	59.87	16.1

*Note:* Mean scores, standard deviations (SD) as well as maximum test score and further calculations for fair comparisons are also included.

Knowledge acquisition is typically assessed through a post‐test administered shortly after the intervention, meaning that the results primarily reflect short‐term acquisition of knowledge by students. The structure of the tests varies among studies (multiple choice questions, OSCEs, written assignments, etc.). Very few studies analyse long‐term retention of knowledge, and some take advantage of nationally regulated tests to provide reliable results. While most studies report their findings in terms of central tendency summaries (mean scores or mean number of correct responses in a test), many studies do not include a summary of variability or dispersion in the data set. Moreover, most studies do not include the number of students who passed or failed the test.

Most included articles found no differences between teaching strategies or a statistically significant difference in favour of the new educational technologies group, while others reported that traditional lectures were the most effective method for knowledge acquisition (Ehsan 2020; Salian et al. 2022; Alsufyani et al. 2023). However, due to the diversity of studies and limitations in the reporting of results for knowledge acquisition, only those studies that reported mean scores and standard deviations (SDs) for both the control and experimental groups, along with the determination of the highest possible test score, were included in the meta‐analysis. Additional inclusion criteria required the presence of two independent experimental groups. Studies in which both groups received the traditional intervention, with or without the innovative format, were excluded from the meta‐analysis. Studies meeting these criteria were then categorised based on the innovative educational intervention for subgroup analysis. Ultimately, 23 articles were included in the global meta‐analysis and were later classified into three distinct categories for subgroup analysis depending on the teaching delivery mode and the structure of the group of students: asynchronous independent learning (individual online flexible learning), (*n* = 5 studies) (Howerton Jr et al. 2004; Maggio et al. 2012; Nkenke et al. 2012; Moazami et al. 2014; Liao et al. 2023), synchronous blended learning (in a large group with the whole class of students) (*n* = 8 articles) (Kavadella et al. 2012; Deepak Nallaswamy et al. 2019; Paul et al. 2019; Isherwood et al. 2020; Naik et al. 2022; Zhong et al. 2023; Iqbal et al. 2024; Varughese et al. 2024) and synchronous small‐group learning (*n* = 7) (Ilgüy et al. 2014; Echeto et al. 2015; Arias et al. 2016; Sagsoz et al. 2017; Rekha et al. 2017; Liu et al. 2020; Guirado et al. 2023). Three studies included in the global meta‐analysis could not be strictly included in any of these three subgroups, as the educational strategies used—such as concept mapping, role‐playing, or a combination of synchronous and asynchronous lectures—did not align with the predefined categories (Kasabah et al. 2016; Messina et al. 2022; Mirzaei et al. 2024).

Student attitudes, satisfaction and perceptions towards the teaching methods were primarily assessed using questionnaires, where students rated their level of agreement with various statements on a Likert scale. In some studies, students were asked to evaluate different teaching strategies or indicate their preferred teaching method. However, in many cases, these surveys were only completed by students in the test group, which makes it challenging to contextualise student satisfaction across the different methods analysed. The studies included in this systematic review consistently showed a clear preference among students for innovative teaching strategies. Students often described these methods as more engaging and noted that they enhanced their learning experience.

### 3.3 Meta‐Analysis

Table 2 shows the characteristics of those studies included in the meta‐analysis by subgroups. Mean scores and SDs, as well as the maximum test score and further calculations for fair comparisons, are also included.

As a first step, since the maximum score varied across studies, the mean and SD of the final test scores were standardised to allow for a fair comparison. The following formulas were used for normalisation:

- Normalised Mean = (Observed Mean/Maximum Score) × 100
- Normalised SD = (Observed SD/Maximum Score) × 100

A random‐effects meta‐analysis was conducted to provide a more generalised estimate of the effect of innovative teaching formats in dental education since the interventions or measured outcomes could differ across studies.

Additionally, subgroup analyses were performed to investigate potential differences in effectiveness based on the type of innovative teaching intervention.

**FIGURE 2:**
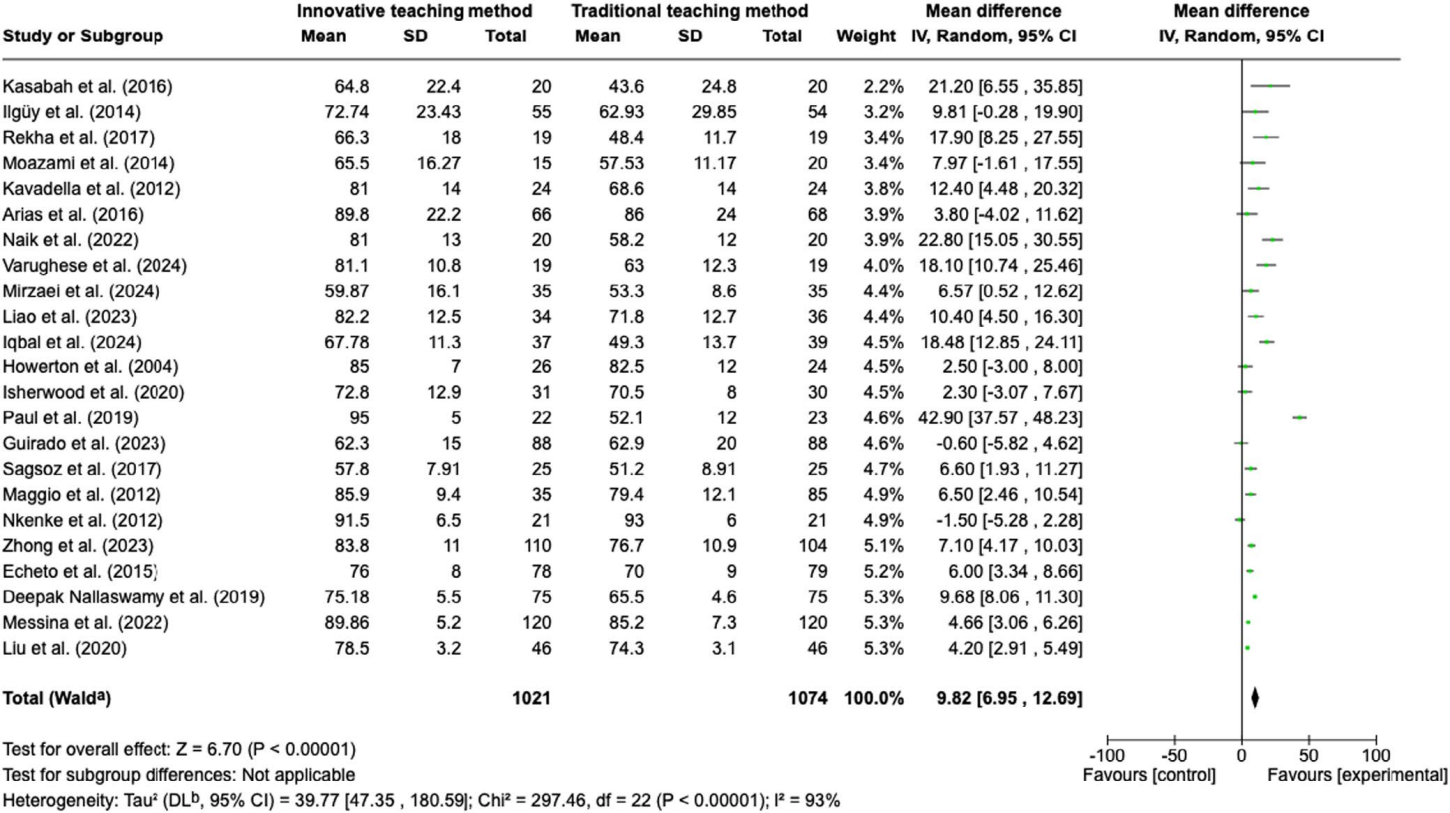
Forest plot for the global group. ^a^CI calculated by Wald‐type method. ^b^Tau^2^ calculated by DerSimonian and Liard method.

**FIGURE 3:**
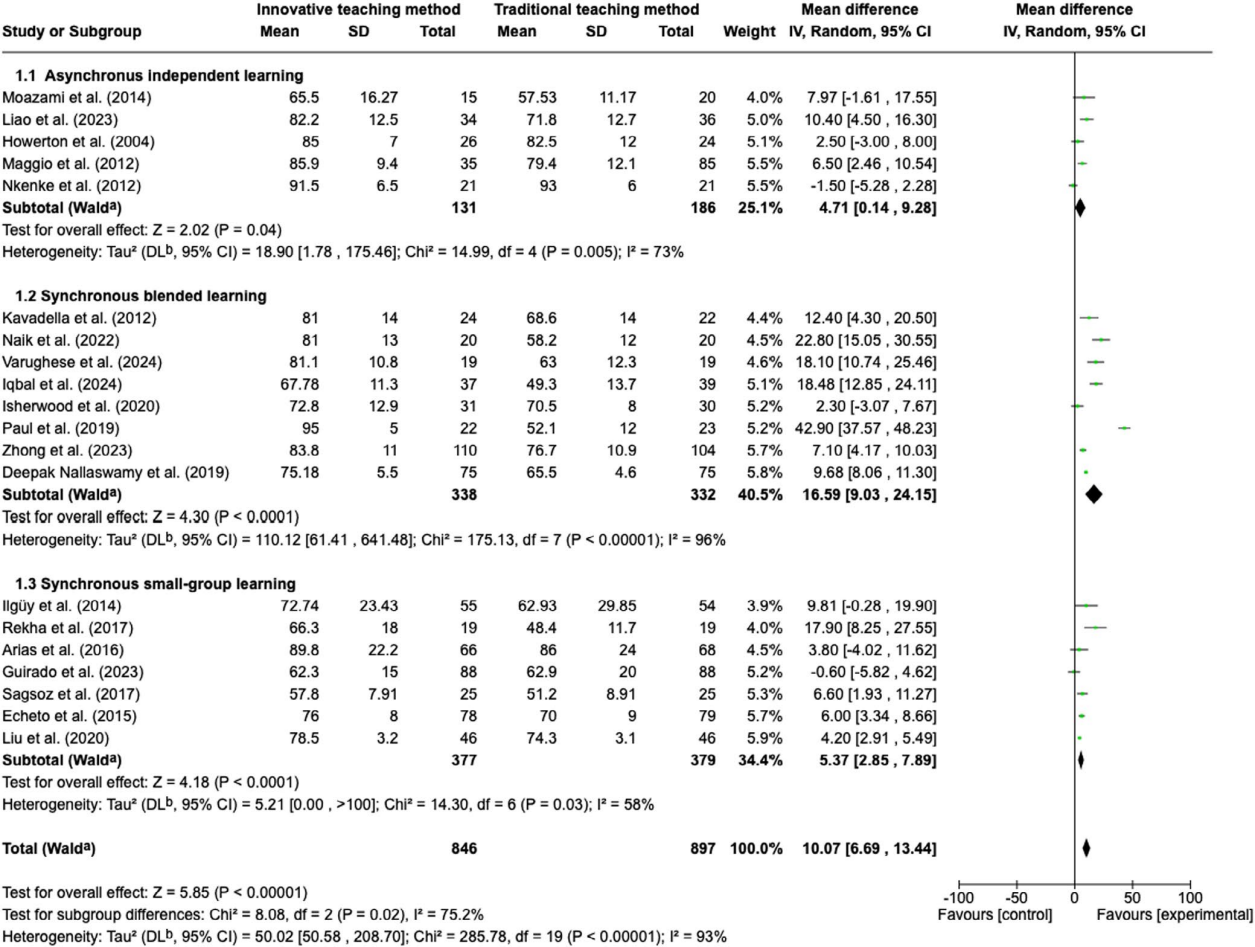
Forest plot for the subgroup analysis. ^a^CI calculated by Wald‐type method. ^b^Tau^2^ calculated by DerSimonian and Liard method.

Figure 2 shows the forest plot for the global group and Figure 3 for the subgroup analysis.

A meta‐analysis of the 23 included studies, encompassing a total of 1074 students in the control and 1021 in the experimental group (Figure 2), revealed significant differences in favour of innovative (experimental) teaching methods (*p* < 0.00001). The overall mean difference was 9.82 (95% confidence interval (CI) = 6.95–12.69) with considerable heterogeneity (*χ* ^2^ = 297.46, *p* < 0.00001; *I* ^2^ = 93%).

Furthermore, the subgroup analysis (Figure 3) also revealed significantly different results depending on the innovative teaching approach (*p* = 0.02). Both asynchronous independent learning and synchronous learning, either in a large group with the whole class of students using blended learning or in small groups, resulted in a significantly better outcome than traditional learning (overall effect: *Z* = 5.85; *p* < 0.00001); however, synchronous blended learning, including techniques like the so‐called FC, showed a significantly better outcome than the rest of the subgroups based on the results of eight studies and 670 students summing both cohorts (mean difference = 16.59; 95% CI = 9.03–24.15) with considerable heterogeneity (*χ* ^2^ = 8.08, *p* = 0.02; *I* ^2^ = 75.2%).

### 3.4 Quality of Evidence

The quality of evidence for the 23 studies included in the meta‐analysis is summarised in Table 3 for randomised controlled trials and Table 4 for non‐randomised studies. As shown in the tables, although significant methodological heterogeneity was observed across studies, only 1 randomised and 2 non‐randomised studies were classified as having a high risk of bias. The primary sources of bias were related to randomisation and outcome measurement (test scores) since some studies did not utilise objective, validated assessment tools or employ blinded evaluators for scoring. Some studies reported test scores without specifying the evaluation process. Additionally, students are allocated into groups following an alphabetical order in some universities, making complete randomisation unfeasible. As a result, those studies employing block randomisation were categorised as having an ‘unclear’ risk of bias in terms of randomisation.

**TABLE 3:** Risk of bias assessment of included randomised studies (RoB).

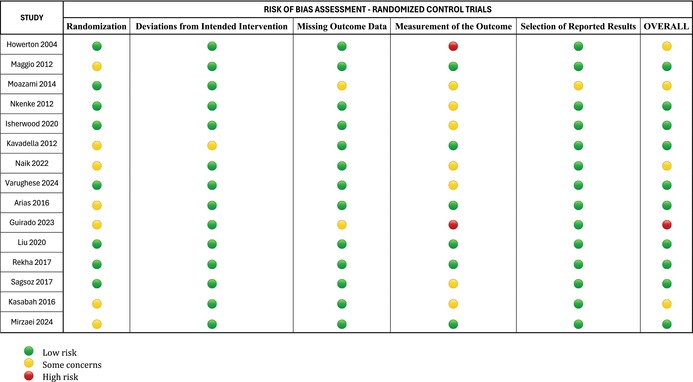

**TABLE 4:** Risk of bias assessment of non‐randomised studies of interventions (ROBINS‐I tool).

Study	Risk of bias assessment—Non‐randomised studies							
	Confounding	Selection of participants	Classification of interventions	Deviations from intended intervention	Missing data	Measurement of outcomes	Selection of reported results	Overall
Liao 2023	Moderate	Low	Low	Moderate	Low	Moderate	Low	Moderate
Deepak Nallaswamy 2019	Moderate	Low	Low	Low	Low	Moderate	Low	Moderate
Iqbal 2024	High	High	Low	Low	Low	Low	Low	High
Paul 2019	High	High	Low	Low	Low	Moderate	Low	High
Zhong 2023	Moderate	Low	Low	Low	Low	Low	Low	Moderate
Echeto 2015	Moderate	Low	Low	Low	Low	Moderate	Low	Moderate
Ilgüy 2014	Moderate	Low	Low	Low	Low	Low	Low	Moderate
Messina 2022	Moderate	Low	Low	Low	Low	Moderate	Low	Moderate

## 4 Discussion

This systematic review and meta‐analysis aimed to evaluate the effectiveness of innovative formats of learning in comparison with traditional formats in terms of educational outcomes and satisfaction. Many studies met the inclusion criteria and were included for further analysis, although with a marked heterogeneity in study design, mostly when assessing students´ perceptions. In terms of knowledge acquisition, 23 studies were selected for further meta‐analysis. Both randomised and non‐randomised studies were combined in the meta‐analysis. Although there are differences in the methodological rigour between randomised and non‐randomised study designs, the COVID‐19 pandemic produced several non‐randomised studies comparing innovative educational methods with previous cohorts and contributing to a better understanding of the effectiveness and evolution of dental education methods when randomisation was not feasible. This was one of the reasons why a random‐effects meta‐analysis was conducted. The approach assumes that the true effect size may vary across studies and allows for the incorporation of both within‐study and between‐study variance. Taken all studies together, the meta‐analysis revealed superior knowledge acquisition with innovative teaching methods than traditional teaching. However, these findings should be interpreted with caution, as none of the included studies assessed long‐term knowledge retention, an essential factor for future practising clinicians. Other review articles in medical education have also highlighted the lack of randomised controlled trials in this field (Considine et al. 2021), yet they also emphasised the positive effects of innovative teaching methods on learning ability, study independence, decision‐making skills and emotional intelligence (Alizadeh et al. 2024).

One of the novelties of the present systematic review was the incorporation of artificial intelligence for duplicate detection and the initial stages of study screening. The use of AI‐powered tools, such as the Rayyan systematic review management platform, might offer several potential advantages. It might help researchers screen, organise and prioritise studies, reducing the time and effort needed for manual screening. The platform facilitates collaboration among multiple reviewers (Ouzzani et al. 2016). However, the role of researchers remains crucial to ensure a fair and unbiased selection of studies, ultimately leading to conclusions that are translatable to university or clinical settings. For this reason, while the app was utilised for an initial screening phase, more screening steps than usual systematic reviews were added to the process to be manually performed by more than two authors to maintain methodological rigour and accuracy.

Regarding the methodology of individual studies included in the systematic review, it should be emphasised that the vast majority explore students´ perceptions as well as short‐term knowledge acquisition. In fact, many studies only assessed immediate knowledge right after the educational activity. Table 4 shows the time allotted to the test exam after the completion of the activity for those studies included in the meta‐analysis. This must be considered when interpreting these findings since an adequate methodology should be capable of providing long‐term retention. There is no consensus as to whether long‐term knowledge retention benefits from new educational strategies (Echeto et al. 2015; Deepak Nallaswamy et al. 2019; Pérez‐Higueras et al. 2024) or whether there is no significant difference among teaching methods (Llena et al. 2018; Godderidge et al. 2019; Fu et al. 2024). The variability in results may be attributed to the absence of standardised time frames for assessing knowledge acquisition in the short, medium, or long term. This lack of consensus on evaluation timelines hinders an effective comparison of educational interventions.

Moreover, many educational studies do not report a minimum set of essential statistical measures, such as summaries of central tendency and dispersion. This inconsistency reduced the number of studies eligible for inclusion in the meta‐analysis. Establishing standardised reporting guidelines could help authors strengthen the rigour and comparability of educational research by reducing the heterogeneity of study designs and improving consistency in reported data. Ultimately, such improvements would facilitate more reliable evidence synthesis, support the development of best practices in educational interventions and contribute to higher‐quality research that can inform teaching and learning in endodontology and other health professions.

At the same time, the self‐perceived acquisition of knowledge or self‐confidence about students´ performance after an educational intervention by means of a questionnaire has also been commonly used to evaluate the effectiveness of different didactic methodologies (Al‐Ahmad 2010; Signori et al. 2019; Puranik et al. 2023). Although the information obtained through this method may provide valuable insights, it does not necessarily reflect the actual knowledge acquisition by students but rather their subjective perception. Nevertheless, an increase in self‐confidence among students has been associated with a more positive attitude towards their future professional careers in dentistry (Signori et al. 2019).

Assessing students' attitudes, satisfaction and perceptions of didactic activities is essential for enhancing educational quality. However, considerable variability has also been observed in the questionnaires used for this purpose. While most authors utilise Likert‐type scales (Kalludi et al. 2015; Ariana et al. 2016; Gerhardt‐Szep et al. 2016; Yakin and Linden 2021; Zhong et al. 2021; Chen et al. 2025), the specific questions included vary significantly across studies, hindering direct comparisons. Additionally, in some cases, surveys are administered exclusively to the test group (Paul et al. 2019; Tuil et al. 2023), making it difficult to accurately assess student satisfaction with traditional educational methods. These findings highlight again the need for a standardised methodology in designing satisfaction and perception surveys. Establishing core aspects to assess student perceptions and formulating uniform questions across studies would facilitate comparability. Furthermore, administering these surveys to all study participants, including both test and control groups, is essential for obtaining comprehensive insights. To address inconsistencies in survey design, guidelines have been proposed to enhance questionnaire quality and ensure methodological rigour (Artino et al. 2014).

Given the large number of studies included, a subgroup meta‐analysis was also feasible. Subgroups were formed by clustering studies that employed similar teaching strategies: asynchronous independent learning, synchronous blended learning in a large group and synchronous collaborative small‐group learning. In all three subgroups, the innovative teaching methods demonstrated superior outcomes in terms of knowledge acquisition compared to traditional learning, with synchronous blended learning with the whole group of students showing a significantly better outcome than the rest of the subgroups.

Positive effects on student satisfaction have been previously reported for synchronous blended learning. The evidence regarding their effectiveness in improving knowledge outcomes had remained limited (Vanka et al. 2020) and was associated with suboptimal utilisation of learning resources provided to students (Mishall et al. 2025). Previous systematic reviews and meta‐analyses have also reported positive outcomes for small‐group learning strategies, emphasising their effectiveness in enhancing student performance (Sharma et al. 2023; Zheng et al. 2023) and fostering critical thinking skills (Sharma et al. 2023; Wei et al. 2024). TBL also enhanced teamwork skills (Zhang et al. 2024). However, several challenges have also been identified, including limited student understanding of the instructional method, inconsistent levels of participation and barriers to implementation, particularly due to differences in student backgrounds (Lin et al. 2025).

The present meta‐analysis also indicates that asynchronous independent learning is associated with improved knowledge acquisition compared to traditional approaches. This finding supports that this type of flexible and accessible (Potomkova et al. 2006; Stevenson et al. 2023) learner‐centred strategies are cost‐effective while offering unrestricted access to learning materials (Kimura et al. 2023) that seem to be particularly engaging if images, videos, or interactive formats are included (Twenge 2009). However, while asynchronous learning promotes autonomy, it also requires students to demonstrate self‐regulation and time management skills (Kimura et al. 2023). These demands are particularly relevant in professional training contexts, where maintaining engagement and motivation remains essential. Therefore, when implementing asynchronous formats, educators should consider incorporating structured guidance and opportunities for interaction to compensate for potential drawbacks related to the reduced social connection and support that exist in face‐to‐face interactions (Sharmin and Chow 2022). Other strategies also showed benefits in the present meta‐analysis. As an example, game‐based learning seems to facilitate the learning process (Sagsoz et al. 2017; Khorasanchi et al. 2024) by improving motivation, satisfaction and collaboration among students (Felszeghy et al. 2019).

Despite the demonstrated benefits of several innovative teaching formats, future research should prioritise the design of studies with robust methodologies, reliable assessments and long‐term follow‐ups in dentistry (Zaror et al. 2021). While some innovative didactic formats seem promising, careful consideration of their implementation and continuous evaluation are crucial for ensuring their effectiveness in dental education. Further well‐designed, randomised controlled trials are needed to provide a more comprehensive understanding of the long‐term impact of these methods on student outcomes.

As a practical application of the findings from this meta‐analysis, the structured integration of innovative teaching strategies such as small‐group learning, blended instruction and asynchronous formats is recommended to improve knowledge acquisition and learner engagement. However, the adoption of these methods must be accompanied by strategic institutional support. Some approaches often require higher professor‐to‐student ratios, increased preparation time for faculty and ongoing professional development to ensure educators are well equipped to design and deliver effective learner‐centred strategies. In addition, strategies like small‐group learning and team‐based activities may require expanded physical spaces to support collaborative learning environments. Aligning curricular innovation with appropriate resources and faculty support is therefore essential to ensure effective implementation.

## 5 Conclusions

This meta‐analysis evaluated the effectiveness of innovative didactic methods compared to traditional teaching approaches in dental education. The overall findings indicate that innovative strategies lead to superior knowledge acquisition in comparison with traditional methods. Subgroup analyses further revealed that both asynchronous independent learning and synchronous learning formats, whether implemented in large‐group settings via blended approaches or in small‐group environments, are more effective than traditional instruction. Among these, synchronous blended learning, including models such as the FC, yields the most favourable outcome. However, the quality of the included studies varied, with some facing methodological challenges such as lack of blinding and inconsistent outcome measurement, which can impact the generalisability of the findings.

## Author Contributions

Conceptualisation: A.A.; methodology, A.A., M.‐S.S., L.G.‐C., I.F.‐G. and J.J.P.‐H.; formal analysis, A.A.; investigation, A.A., M.‐S.S., L.G.‐C., I.F.‐G., J.J.P.‐H.; data curation, A.A., I.F.‐G. and J.J.P.‐H.; writing – original draft preparation, A.A., M.‐S.S., L.G.‐C., I.F.‐G. and J.J.P.‐H.; writing – review and editing, A.A.; supervision, A.A., J.J.P.‐H. All authors have read and agreed to the published version of the manuscript.

## Conflicts of Interest

The authors declare no conflicts of interest.

## Supporting information


**Table S1:** iej70006‐sup‐0001‐TableS1.docx.

## Data Availability

The data that supports the findings of this study are available in the Supporting Information of this article.
