# Supplementary material for: Innovative Didactic Learning Formats: Have They Improved Dental Education? A Systematic Review and Meta‐Analysis
Source: Int Endod J. 2025 Aug 28;59(6):943–67. doi: 10.1111/iej.70006 (PMC13158554; doi:10.1111/iej.70006)
Supplement: Supplementary file 1 — Table S1: iej70006‐sup‐0001‐TableS1.docx. [file IEJ-59-943-s001.docx]

| **REASON** | **authors** | **year** | **journal** |
| --- | --- | --- | --- |
| Book Chapter | Nikolaidis N et al. | 2009 | Dental Computing and Applications: Advanced Techniques for Clinical Dentistry |
|  | Shuler CF | 2012 | Problem-Based Learning in Clinical Education: The Next Generation |
|  | Vagg T et al. | 2023 | Biomedical Visualisation, Vol. 15: Visualisation in Teaching of Biomedical and Clinical Subjects: Anatomy, Advanced Microscopy and Radiology Advanced Microscopy and Radiology |
| Development and/or implementation of 1 methodology | Alkatheri AM et al. | 2018 | Health Professions Education |
|  | Allen T et al. | 2022 | Health Promotion Journal of Australia |
|  | Anders PL et al. | 2016 | Journal of Dental Education |
|  | Anderson P et al. | 2014 | Intelligent Systems Reference Library |
|  | Bakr MM et al. | 2017 | Anatomical Sciences Education |
|  | Barac'h V et al. | 2001 | Studies in Health Technology and Informatics |
|  | Binnie RSL and Bonsor SJ | 2021 | British Dental Journal |
|  | Bogdan CM and Popovici DM | 2012 | Computer Methods and Programs in Biomedicine |
|  | Bokhari SMA and Khan SA | 2016 | Studies in Computational Intelligence |
|  | Bridges S | 2015 | Pedagogies |
|  | Broder HL et al. | 2015 | Journal of Dental Education |
|  | Brondani MA and Rossoff LP | 2010 | Journal of Dental Education |
|  | Broudo M and Walsh C | 2002 | Academic Medicine |
|  | Brown T et al. | 2021 | MedEdPORTAL The Journal of Teaching and Learning Resources |
|  | Buchanan JA | 2004 | Journal of Dental Education |
|  | Campbell F and Rogers H | 2024 | British Dental Journal |
|  | Castillo J et al. | 2017 | Journal of Medical Education and Curricular Development |
|  | Chambers DW | 2009 | Journal of Dental Education |
|  | Chen AK et al. | 2017 | Journal of Interprofessional Education and Practice |
|  | Chen M-L et al. | 2011 | Journal of Medical Systems |
|  | Cohen HB et al. | 2003 | Journal of Dental Education |
|  | Cohen SN and Silvestri Jr AR | 1979 | Journal of Dental Education |
|  | Corrêa L et al. | 2003 | European Journal of Dental Education |
|  | Crothers AJ et al. | 2017 | British Dental Journal |
|  | Darby M | 2007 | Journal of Dental Hygiene |
|  | de Boer IR et al. | 2018 | European Journal of Dental Education |
|  | de Peralta TL et al. | 2019 | Journal of Dental Education |
|  | de Shazer DO | 1971 | Journal of Dental Education |
|  | Deogade SC and Naitam D | 2016 | Education for Health: Change in Learning and Practice |
|  | Emling RC and Gellin ME | 1975 | Journal of Dental Education |
|  | Emling RC and Gellin ME | 1976 | Journal of Dental Education |
|  | Esaulenko IE et al. | 2018 | Research Journal of Pharmaceutical Biological and Chemical Sciences |
|  | Esquer FG et al. | 2009 | REDU - Revista de Docencia Universitaria |
|  | Feng J et al. | 2021 | Journal of Dental Education |
|  | Fouillen KJ et al. | 2023 | International Endodontic Journal |
|  | Foxton R et al. | 2023 | European Journal of Dental Education |
|  | France K et al. | 2021 | Journal of Dental Education |
|  | Gadbury-Amyot C and Brockman WG | 2011 | Journal of Dental Education |
|  | Gatt G and Attard NJ | 2023 | BMC Medical Education |
|  | Gibbard LL and Salajan F | 2009 | Electronic Journal of E-Learning |
|  | Goodacre CJ | 2018 | Journal of Prosthodontics |
|  | Goto T et al. | 2019 | Clinical and Experimental Dental Research |
|  | Gredes T et al. | 2022 | Journal of Dental Education |
|  | Gu J-Y and Lee J-G | 2019 | Journal of Information and Communication Convergence Engineering |
|  | Hadyaoui D et al. | 2024 | Journal of Contemporary Dental Practice |
|  | Himida T et al. | 2019 | European Journal of Dental Education |
|  | Höhne C et al. | 2019 | Journal of Dental Education |
|  | Höhne S and Schumann RR | 2004 | International Journal of Computerized Dentistry |
|  | Hu L et al. | 2023 | European Journal of Dental Education |
|  | Im J-E Gu J-Y et al. | 2023 | Virtual Reality |
|  | Jakab J et al. | 2021 | Collegium Antropologicum |
|  | Jannot M et al. | 2024 | Journal of Dental Education |
|  | Khorasanchi M et al. | 2024 | BMC Medical Education |
|  | Klink A et al. | 2024 | Dentistry Journal |
|  | Kruppke B | 2021 | Education Sciences |
|  | Lara JS et al. | 2020 | JMIR Medical Education |
|  | Legorburu BG et al. | 2022 | Bioengineering |
|  | Li Y et al. | 2022 | JMIR Serious Games |
|  | Lone M et al. | 2018 | European Journal of Dental Education |
|  | McGoldrick PM and Pine CM | 2000 | European Journal of Dental Education |
|  | Naser-ud-Din S | 2015 | European Journal of Dental Education |
|  | Nizami MZI et al. | 2023 | Dentistry Journal |
|  | Omar H et al. | 2021 | Journal of Dental Education |
|  | Park K et al. | 2024 | Journal of Dental Education |
|  | Park SE and Howell TH | 2015 | Journal of Dental Education |
|  | Park SE et al. | 2019 | European Journal of Dental Education |
|  | Park SE et al. | 2012 | Journal of Dental Education |
|  | Risnes S et al. | 2019 | European Journal of Dental Education |
|  | Riutord-Sbert P et al. | 2023 | Medicina Balear |
|  | Rosenbaum P-EL et al. | 2012 | Open Dentistry Journal |
|  | Rosenberg H et al. | 2010 | American Journal of Orthodontics and Dentofacial Orthopedics |
|  | Rubin RW et al. | 2008 | Journal of Dental Education |
|  | Sanchez SJM et al. | 2019 | CSEDU - Proceedings of the 11th International Conference on Computer Supported Education |
|  | Sanders C et al. | 2008 | Special Care in Dentistry |
|  | Saunders TR and Dejbakhsh S | 2007 | Journal of Prosthodontics |
|  | Sharmin N et al. | 2022 | Healthcare Informatics Research |
|  | Sherif M et al. | 2023 | ICCA - 5th International Conference on Computer and Applications, Proceedings |
|  | Shiloah J et al. | 2017 | European Journal of Dental Education |
|  | Shrestha B et al. | 2023 | Journal of Nepal Health Research Council |
|  | Shuler CF | 2002 | Journal of the California Dental Association |
|  | Shuler CF | 2001 | The Journal of the American College of Dentists |
|  | Siddanna G et al. | 2024 | Oral |
|  | Stelzle F et al. | 2011 | Acta Odontologica Scandinavica |
|  | Teerawongpairoj C et al. | 2024 | Scientific Reports |
|  | Tsai R et al. | 2020 | Journal of Taibah University Medical Sciences |
|  | Veeraiyan DN and Sekhar P | 2013 | Journal of Dental Education |
|  | Veeraiyan DN et al. | 2022 | International Journal of Early Childhood Special Education |
|  | Veses V et al. | 2020 | BMC Medical Education |
|  | Victorelli G et al. | 2014 | Journal of Dental Education |
|  | Vuchkova J et al. | 2012 | European Journal of Dental Education |
|  | Wagner J et al. | 2007 | Journal of Dental Education |
|  | Wagner JA et al. | 2011 | Evaluation and the Health Professions |
|  | Walker J and von Bergmann H | 2015 | Journal of Dental Education |
|  | Wang HL et al. | 2020 | BMC Medical Education |
|  | Wang W et al. | 2020 | PeerJ |
|  | Weiner CK et al. | 2016 | Journal of Dental Education |
|  | Wener ME et al. | 2011 | Journal of Dental Education |
|  | Werz SM et al. | 2018 | European Journal of Dental Education |
|  | Wiener RC et al. | 2018 | Journal of Dental Education |
|  | Wu J-H et al. | 2021 | Medical Education Online |
|  | Wu J-H et al. | 2024 | BMC Medical Education |
|  | Wuenschell CW et al. | 2007 | Journal of Dental Education |
|  | Yari A et al. | 2024 | Journal of Dental Education |
|  | Yeng T et al. | 2022 | Australian Endodontic Journal |
|  | Yoshida N et al. | 2012 | International Journal of Dental Hygiene |
|  | Yoshida T et al. | 2014 | European Journal of Dental Education |
|  | Yoshida Y et al. | 2011 | Dental Materials Journal |
|  | Young A et al. | 2022 | Caries Research |
|  | Yui T et al. | 2022 | IEEE Access |
|  | Zaug P et al. | 2022 | European Journal of Dental Education |
|  | Zeng Y et al. | 2024 | European Journal of Dental Education |
|  | Zhang R et al. | 2019 | BMC Medical Education |
| Implementation or assessment of curriculum / program / module | Abe Y et al. | 2021 | Journal of Dental Education |
|  | Al-Zubaidi SM et al. | 2024 | Cureus Journal of Medical Science |
|  | Gregson K et al. | 2010 | Journal of Dental Education |
|  | Hattar S et al. | 2020 | International Journal of Dentistry |
|  | Hoad-Reddick G and Theaker E | 2003 | European Journal of Dental Education |
|  | Iacopino AM et al. | 2007 | Journal of Dental Research |
|  | Inayat N et al. | 2021 | Pakistan Journal of Medical and Health Sciences |
|  | Ingebrigtsen J et al. | 2008 | European Journal of Dental Education |
|  | Kanzow P et al. | 2021 | JMIR Medical Education |
|  | Katsuragi H | 2005 | Odontology |
|  | Khanna R et al. | 2021 | Medical Journal Armed Forces India |
|  | Kohli S et al. | 2023 | Cumhuriyet Dental Journal |
|  | Koth AJ et al. | 2021 | Journal of Dental Education |
|  | Krüger C et al. | 2005 | South African Family Practice |
|  | Kumar P et al. | 2020 | Journal of Education and Health Promotion |
|  | Leary S and Ness A | 2021 | Journal of University Teaching and Learning Practice |
|  | Leary S and Davies A | 2023 | Teaching Biostatistics in Medicine and Allied Health Sciences |
|  | Lennon AM et al. | 2001 | Journal of Dental Education |
|  | Leong C et al. | 2015 | Journal of Interprofessional Care |
|  | Lin GSS and Foong CC | 2024 | European Journal of Dental Education |
|  | Lockhart DEA and Smith AJ | 2009 | British Dental Journal |
|  | Marshall TA et al. | 2011 | Journal of Dental Education |
|  | Marti K et al. | 2019 | Journal of Dental Education |
|  | Mather C et al. | 2023 | Advances in Experimental Medicine and Biology |
|  | Mitchell J and Brackett M | 2017 | MedEdPORTAL The Journal of Teaching and Learning Resources |
|  | Mohebbi SZ et al. | 2014 | European Journal of Dental Education |
|  | Morales-Pérez MA et al. | 2020 | Journal of Dental Education |
|  | Möst T et al. | 2013 | European Journal of Dental Education |
|  | Nallaswamy VD et al. | 2022 | International Journal of Early Childhood Special Education |
|  | Obrez A et al. | 2011 | Journal of Dental Education |
|  | Obrez A et al. | 2009 | Journal of Dental Education |
|  | Oetter N et al. | 2022 | BMC medical education |
|  | Oh SL et al. | 2024 | Journal of Dental Education |
|  | Petersson K et al. | 2002 | European Journal of Dental Education |
|  | Poudel P and Adhikari BR | 2024 | Kathmandu University Medical Journal |
|  | Ramseier CA et al. | 2024 | European Journal of Dental Education |
|  | Richards C et al. | 2024 | European Journal of Dental Education |
|  | Rohlin M et al. | 1998 | European Journal of Dental Education |
|  | Springfield EC et al. | 2015 | Journal of Dental Education |
|  | Susarla SM et al. | 2003 | Journal of Dental Education |
|  | Tantawi MMAE | 2009 | Journal of Dental Education |
|  | Teich ST et al. | 2015 | Journal of Dental Education |
|  | Vahed A and Rodriguez K | 2020 | Innovations in Education and Teaching International |
|  | Winning T et al. | 2005 | Assessment and Evaluation in Higher Education |
|  | Wu J et al. | 2016 | Journal of Dental Education |
|  | Yu A et al. | 2020 | Journal of Dental Education |
| No article format / Short communications / Editorials | Almarzouqi A et al. | 2024 | Proceedings - IEEE 48th Annual Computers, Software, and Applications Conference, COMPSAC |
|  | Beltes C et al. | 2024 | Digital Dentistry: An Overview and Future Prospects |
|  | Berlin-Broner Y and Levin L | 2019 | Quintessence International |
|  | Braga MM et al. | 2017 | JMIR Research Protocols |
|  | Carreon M et al. | 2020 | ACM International Conference Proceeding Series |
|  | Cheng Z et al. | 2024 | Lecture Notes in Computer Science (including subseries Lecture Notes in Artificial Intelligence and Lecture Notes in Bioinformatics) |
|  | Chockalingam S and Sandeep H | 2023 | 2nd International Conference on Business Analytics for Technology and Security, ICBATS |
|  | Cox MJ et al. | 2017 | IFIP Advances in Information and Communication Technology |
|  | Crawford S et al. | 2022 | Proceedings - IEEE Conference on Virtual Reality and 3D User Interfaces Abstracts and Workshops, VRW |
|  | Higgins D et al. | 2020 | European Journal of Dental Education |
|  | Hupp JR | 2019 | Journal of Oral and Maxillofacial Surgery |
|  | Kamboj M et al. | 2010 | Journal of Dental Education |
|  | Moxham BJ and Pais D | 2017 | Clinical Anatomy |
|  | Poly A et al. | 2024 | Journal of Dental Education |
|  | Singh A and Mills S | 2023 | Journal of Dental Education |
|  | Singh A et al. | 2024 | Journal of Dental Education |
|  | Tao CY et al. | 2023 | Journal of Dental Education |
|  | Varghese SS et al. | 2019 | Journal of Dental Education |
| No comparison between/among different educational methods | Abdalla R | 2020 | European Journal of Dental Education |
|  | Abdelaal HM et al. | 2023 | Journal of International Dental and Medical Research |
|  | Abdelkarim A et al. | 2018 | Journal of Dental Education |
|  | Aboalshamat K et al. | 2015 | BMC Medical Education |
|  | Acharya S et al. | 2021 | Pesquisa Brasileira em Odontopediatria e Clinica Integrada |
|  | Afrashtehfar KI et al. | 2023 | F1000Research |
|  | Ahmad M et al. | 2016 | Journal of Dental Education |
|  | Ahmed F and Sharma PK | 2023 | Journal of Orthodontics |
|  | Ahmed S et al. | 2023 | Journal of the Pakistan Medical Association |
|  | Akbar Z et al. | 2023 | Pakistan Armed Forces Medical Journal |
|  | Akhlaghi N et al. | 2018 | Journal of Educational Evaluation for Health Professions |
|  | Al Ehaideb A et al. | 2024 | Saudi Dental Journal |
|  | Al Kawas S and Hamdy H | 2017 | Health Professions Education |
|  | Al-Jandan BA et al. | 2015 | Journal of Taibah University Medical Sciences |
|  | Al-Khalifa KS and Gaffar BO | 2021 | Journal of Dental Education |
|  | Al-Khalifa KS and Nazir MA | 2020 | Journal of Taibah University Medical Sciences |
|  | Al-Rawi W et al. | 2015 | Journal of Dental Education |
|  | Al-Sebaei MO | 2023 | BMC Medical Education |
|  | Al-Shaikh GK et al. | 2018 | Medical Teacher |
|  | Al-Shamrani SS et al. | 2021 | Bioscience Biotechnology Research Communications |
|  | Al-Taweel FB et al. | 2021 | European Journal of Dental Education |
|  | AlAhmari F | 2022 | Journal of Research in Medical and Dental Science |
|  | Alfadley A et al. | 2020 | Saudi Endodontic Journal |
|  | Alfouzan AF et al. | 2017 | Journal of Esthetic and Restorative Dentistry |
|  | Ali K et al. | 2023 | Medical Education Online |
|  | Ali K et al. | 2024 | European Journal of Dental Education |
|  | Ali K et al. | 2023 | European Journal of Dental Education |
|  | Ali K et al. | 2016 | Journal of Dental Education |
|  | Ali T and Aziz S | 2015 | Annals Abbasi Shaheed Hospital & Karachi Medical & Dental College |
|  | Alkahtany SM | 2020 | International Journal of Medical Dentistry |
|  | Alkhuwaiter S et al. | 2016 | Journal of International Society of Preventive and Community Dentistry |
|  | Allaire JL | 2015 | Journal of Dental Education |
|  | Alqaderi H et al. | 2019 | Journal of Dental Education |
|  | Alroomy R et al. | 2024 | European Endodontic Journal |
|  | Alshiekhly U et al. | 2015 | Education for Health: Change in Learning and Practice |
|  | Amin IM et al. | 2013 | BEIAC - IEEE Business Engineering and Industrial Applications Colloquium |
|  | Amir LR et al. | 2020 | BMC Medical Education |
|  | Anderson V and Reid K | 2012 | European Journal of Dental Education |
|  | Arayapisit T et al. | 2023 | Anatomical Sciences Education |
|  | Arigbede A et al. | 2015 | African Health Sciences |
|  | Arroyo-Bote S et al. | 2024 | Journal of Dental Education |
|  | Asiry MA | 2017 | Saudi Dental Journal |
|  | Atiah N et al. | 2024 | Journal of Dental Education |
|  | Aubeux D et al. | 2020 | European Journal of Dental Education |
|  | Aurora F et al. | 2023 | British Journal of Oral and Maxillofacial Surgery |
|  | Awad Z et al. | 2023 | Oral Surgery |
|  | Awasthi R et al. | 2022 | Advances in Human Biology |
|  | Azeem M et al. | 2018 | Pakistan Journal of Medical and Health Sciences |
|  | Ba-Hattab R et al. | 2023 | Applied Sciences (Switzerland) |
|  | Badovinac A et al. | 2021 | Dentistry Journal |
|  | Baechle MA et al. | 2022 | Journal of Dental Education |
|  | Bagewadi A | 2021 | Journal of Indian Academy of Oral Medicine and Radiology |
|  | Bahanan L et al. | 2022 | Advances in Medical Education and Practice |
|  | Baherimoghadam T et al. | 2021 | BMC Medical Education |
|  | Balhaddad AA et al. | 2021 | European Journal of Dental Education |
|  | Barbour ME | 2008 | Journal of Dental Education |
|  | Barker TS et al. | 2018 | Journal of Dental Hygiene |
|  | Barman A et al. | 2006 | Malaysian Journal of Medical Sciences |
|  | Barman A et al. | 2006 | Education for Health: Change in Learning and Practice |
|  | Bartok-Nicolae C et al. | 2022 | Applied Sciences (Switzerland) |
|  | Bartolomé Villar B et al. | 2022 | International Journal of Environmental Research and Public Health |
|  | Bearn DR and Chadwick SM | 2010 | European Journal of Dental Education |
|  | Beattie BE et al. | 2014 | Journal of Dental Education |
|  | Becker T et al. | 2024 | GMS Journal for Medical Education |
|  | Bedi R et al. | 2002 | Primary Dental Care Journal of the Faculty of General Dental Practitioners (UK) |
|  | Behar-Horenstein LS and Feng X | 2017 | Qualitative Report |
|  | Berry M et al. | 2022 | European Journal of Dental Education |
|  | Bharath C et al. | 2022 | Journal of Pharmacy and Bioallied Sciences |
|  | Bizhang M et al. | 2022 | International Journal of Dentistry |
|  | Bock A et al. | 2020 | European Journal of Dental Education |
|  | Bock A et al. | 2024 | BMC Medical Education |
|  | Bohaty BS et al. | 2016 | Journal of Dental Education |
|  | Bompolaki D and Stafford G | 2023 | European Journal of Dental Education |
|  | Botelho M and Bhuyan SY | 2021 | European Journal of Dental Education |
|  | Botelho MG | 2019 | Journal of Investigative and Clinical Dentistry |
|  | Botelho MG | 2019 | European Journal of Dental Education |
|  | Botelho MG et al. | 2023 | European Journal of Dental Education |
|  | Botelho MG and Chan AKM | 2022 | European Journal of Dental Education |
|  | Botelho MG et al. | 2019 | European Journal of Dental Education |
|  | Botelho MG et al. | 2013 | European Journal of Dental Education |
|  | Bowers RD et al. | 2022 | Dentistry Journal |
|  | Bridges S et al. | 2014 | European Journal of Dental Education |
|  | Brumini G et al. | 2014 | European Journal of Dental Education |
|  | Burns LE et al. | 2020 | Journal of Dental Education |
|  | Caussin É et al. | 2024 | European Journal of Dental Education |
|  | Chan AKM et al. | 2021 | European Journal of Dental Education |
|  | Chandu VC et al. | 2021 | Population Medicine |
|  | Chao J et al. | 2024 | Journal of Dental Education |
|  | Chauhan A and Angadi PV | 2024 | Annals of Dental Specialty |
|  | Chen D et al. | 2022 | Clinical and Experimental Pharmacology and Physiology |
|  | Cho A and Ganesh N | 2022 | Journal of Dental Education |
|  | Choi S et al. | 2023 | European Journal of Dental Education |
|  | Chutinan S et al. | 2021 | European Journal of Dental Education |
|  | Clark B et al. | 2012 | Clinical Teacher |
|  | Codeço A et al. | 2020 | European Journal of Dental Education |
|  | Coro-Montanet G et al. | 2022 | International Journal of Environmental Research and Public Health |
|  | Costa ED et al. | 2022 | Odovtos - International Journal of Dental Sciences |
|  | Costa ST et al. | 2022 | Revista Ibero-Americana de Estudos em Educaçao |
|  | Croft P et al. | 2005 | European Journal of Dental Education |
|  | da Costa-Neri AR et al. | 2022 | Acta Odontologica Latinoamericana |
|  | da Silva LRG et al. | 2023 | Brazilian Oral Research |
|  | Dascalu CG et al. | 2023 | Applied Sciences (Switzerland) |
|  | Dascalu CG et al. | 2021 | Revista de Cercetare Si Interventie Sociala |
|  | Daud A et al. | 2024 | BMC Oral Health |
|  | De Boer IR et al. | 2019 | Simulation in Healthcare |
|  | de Pedro M et al. | 2022 | European Journal of Dental Education |
|  | Deepak Nallaswamy V et al. | 2019 | International Journal of Research in Pharmaceutical Sciences |
|  | Demirel A et al. | 2022 | Cumhuriyet Dental Journal |
|  | Dhahri AA et al. | 2021 | Journal of Pharmaceutical Research International |
|  | Dhanabal N et al. | 2021 | Journal of Young Pharmacists |
|  | Dias da Silva MA et al. | 2022 | European Journal of Dental Education |
|  | Diniz MB et al. | 2010 | Journal of Dental Education |
|  | Drahos GL | 2017 | Australasian Medical Journal |
|  | Duś-Ilnicka I et al. | 2024 | BMC Medical Education |
|  | Dyulicheva YYu et al. | 2021 | CEUR Workshop Proceedings |
|  | Ehsan A et al. | 2023 | Journal of University Medical and Dental College |
|  | El Tantawi M et al. | 2018 | European Journal of Dental Education |
|  | El Tantawi MMA et al. | 2014 | Journal of Dental Education |
|  | El Tantawi MMA et al. | 2015 | Journal of Dental Education |
|  | El-Damanhoury HM et al. | 2014 | European Journal of Dentistry |
|  | Escobar A et al. | 2022 | Journal of Dental Education |
|  | Escudier MP et al. | 2018 | European Journal of Dental Education |
|  | Fahim A et al. | 2023 | Journal of Dental Education |
|  | Farag A and Hashem D | 2022 | Clinics and Practice |
|  | Farah CS and Maybury TS | 2009 | Journal of Dental Education |
|  | Faraone KL et al. | 2013 | European Journal of Dental Education |
|  | Farghal NS et al. | 2023 | Journal of Contemporary Dental Practice |
|  | Fasbinder DJ et al. | 2015 | Journal of Dental Education |
|  | Feil P | 1992 | Journal of Dental Education |
|  | Ferguson DB and Rutishauser SCB | 1997 | British Dental Journal |
|  | Fincham AG et al. | 1997 | Journal of Dental Education |
|  | Fischer KM et al. | 2024 | Journal of Dental Education |
|  | Fischer T et al. | 2024 | European Journal of Dental Education |
|  | Forni R et al. | 2024 | Journal of Dental Education |
|  | Fu M-W et al. | 2022 | Journal of Dental Education |
|  | Fuhrmann S et al. | 2022 | European Journal of Dental Education |
|  | Gadbury-Amyot CC et al. | 2013 | Journal of Dental Education |
|  | Galibourg A et al. | 2024 | European Journal of Dental Education |
|  | Gallagher JE et al. | 2024 | European Journal of Dental Education |
|  | Ganji KK et al. | 2023 | Healthcare (Switzerland) |
|  | Gao X et al. | 2015 | European Journal of Dental Education |
|  | Gil YM and Lee B-D | 2024 | Journal of Dental Education |
|  | Gilani R et al. | 2020 | Journal of Datta Meghe Institute of Medical Sciences University |
|  | Goertzen E et al. | 2023 | Oral Surgery, Oral Medicine, Oral Pathology and Oral Radiology |
|  | González-Carrasco D et al. | 2022 | Medicina Balear |
|  | Gonzalez SM and Gadbury-Amyot CC | 2016 | Journal of Dental Education |
|  | Gor I et al. | 2021 | International Journal of Web-Based Learning and Teaching Technologies |
|  | Grady R et al. | 2009 | European Journal of Dental Education |
|  | Haj-Ali R and Al Quran F | 2013 | Journal of Dental Education |
|  | Hasan SJ et al. | 2024 | Internet Journal of Allied Health Sciences and Practice |
|  | Hawley N et al. | 2009 | Journal of Dental Education |
|  | Hayashi MR et al. | 2018 | Journal of Dental Education |
|  | Hayes C et al. | 2022 | Journal of Dental Education |
|  | Hegde AM et al. | 2023 | Journal of Health and Allied Sciences Un |
|  | Hinz JG | 2010 | Journal of Dental Education |
|  | Ho ACH et al. | 2022 | European Journal of Dental Education |
|  | Höhne C et al. | 2020 | European Journal of Dental Education |
|  | Hum LN et al. | 2024 | Journal of Dental Education |
|  | Hunt T et al. | 2020 | Journal of Dental Education |
|  | Ihm J et al. | 2017 | Korean Journal of Medical Education |
|  | Ihm J et al. | 2020 | Journal of Dental Education |
|  | Inquimbert C et al. | 2019 | European Journal of Dentistry |
|  | Jackson SC et al. | 2018 | Journal of Dental Education |
|  | Jaganathan S et al. | 2020 | Journal of Pharmacy and Bioallied Sciences |
|  | Johnsen J-AK et al. | 2023 | European Journal of Dental Education |
|  | Johnson JT | 2005 | Journal of Dental Education |
|  | Jones VE et al. | 2017 | Journal of Dental Education |
|  | Joseph D et al. | 2023 | European Journal of Dental Education |
|  | Joseph D et al. | 2014 | BioMed Research International |
|  | Kahatab AF et al. | 2018 | European Journal of Dental Education |
|  | Kalaigian AW and Klein BA | 2024 | Journal of Dental Education |
|  | Kaluschke M et al. | 2023 | PLoS ONE |
|  | Karandish M | 2020 | Journal of Dental Education |
|  | Karandish M et al. | 2024 | Clinical and Experimental Dental Research |
|  | Karapinar-Kazandag M et al. | 2018 | Biomedical Research (India) |
|  | Khan SA et al. | 2012 | Journal of Dental Education |
|  | Khattak O et al. | 2024 | Cureus Journal of Medical Science |
|  | Khayyam L et al. | 2019 | Journal of Medical Education Development |
|  | Kim-Berman H et al. | 2023 | Journal of Dental Education |
|  | Kim A | 2020 | MedEdPORTAL The Journal of Teaching and Learning Resources |
|  | Kingsley K et al. | 2007 | Journal of Dental Education |
|  | Kishimoto N et al. | 2018 | European Journal of Dental Education |
|  | Kniha K et al. | 2023 | European Journal of Dental Education |
|  | Knipfer C et al. | 2018 | BMC Medical Education |
|  | Kolling M et al. | 2022 | European Journal of Dental Education |
|  | Kon H et al. | 2017 | Journal of Investigative and Clinical Dentistry |
|  | Koole S et al. | 2014 | Journal of Dental Education |
|  | Krumsvik RJ et al. | 2020 | Designs for Learning |
|  | Kruse AB et al. | 2023 | GMS Journal for Medical Education |
|  | Lahoti KS et al. | 2021 | Journal of Pharmaceutical Research International |
|  | Lalla RV et al. | 2019 | Journal of Dental Education |
|  | Lee M et al. | 2024 | Journal of Medical Internet Research |
|  | Leisnert L et al. | 2012 | European Journal of Dental Education |
|  | Lin C-S and Yang C-C | 2023 | BMC Medical Education |
|  | Lin GSS and Chua KH | 2024 | Educacion Medica |
|  | Lin GSS et al. | 2023 | European Journal of Dental Education |
|  | Linjawi A and Agou S. | 2020 | Journal of Microscopy and Ultrastructure |
|  | Linjawi AL et al. | 2009 | European Journal of Dental Education |
|  | Liu L et al. | 2020 | European Journal of Dental Education |
|  | Lopez EKN et al. | 2022 | Journal of Dental Education |
|  | Lund B et al. | 2011 | BMC Medical Education |
|  | Luo F et al. | 2024 | BMC Medical Education |
|  | Luo J et al. | 2022 | Disease Markers |
|  | Mascarenhas S et al. | 2021 | Korean Journal of Medical Education |
|  | Matsuka Y et al. | 2012 | Journal of Dental Education |
|  | McAndrew M et al. | 2016 | Journal of Dental Education |
|  | McKenzie CT | 2013 | Journal of Dental Education |
|  | Meschi M, Shirahmadi S et al. | 2024 | BMC Medical Education |
|  | Mirghani I et al. | 2018 | European Journal of Dental Education |
|  | Mitov G et al. | 2010 | Journal of Dental Education |
|  | Mohammed CA et al. | 2021 | European Journal of Dental Education |
|  | Mohd Suria TYI et al. | 2023 | Special Care in Dentistry |
|  | Möser M et al. | 2023 | GMS Journal for Medical Education |
|  | Mulla SA et al. | 2023 | European Journal of General Dentistry |
|  | Mumtaz S and Sabir S | 2022 | Cureus Journal of Medical Science |
|  | Nagata JY et al. | 2018 | European Journal of Dentistry |
|  | Naguib GH et al. | 2021 | Bioscience Biotechnology Research Communications |
|  | Nanda MS et al. | 2020 | Journal of Evolution of Medical and Dental Sciences |
|  | Nassief S et al. | 2024 | Journal of Dental Education |
|  | Nguyen LM et al. | 2023 | Journal of Dental Education |
|  | Nijakowski K et al. | 2021 | International Journal of Environmental Research and Public Health |
|  | Nishigawa K et al. | 2017 | Journal of Dental Education |
|  | Numasawa M et al. | 2021 | PLoS ONE |
|  | Ohsato A et al. | 2022 | Journal of Dental Sciences |
|  | Palcanis KG et al. | 2012 | Journal of Dental Education |
|  | Pang L et al. | 2024 | BMC Medical Education |
|  | Pang M et al. | 2022 | JMIR Serious Games |
|  | Panhwar M et al. | 2021 | Rawal Medical Journal |
|  | Papadopoulos L et al. | 2013 | Journal of Medical Internet Research |
|  | Pardamean B | 2012 | Journal of Dental Education |
|  | Parolia A et al. | 2012 | Journal of Dental Education |
|  | Partido BB et al. | 2020 | Journal of Dental Education |
|  | Pasupuleti MK et al. | 2024 | Frontiers of Oral and Maxillofacial Medicine |
|  | Patel S et al. | 2024 | Journal of Dental Education |
|  | Pawlaczyk-Kamieńska T et al. | 2023 | International Journal of Environmental Research and Public Health |
|  | Perez A et al. | 2022 | Journal of Dental Education |
|  | Perez A et al. | 2023 | European Journal of Dental Education |
|  | Persky AM et al. | 2017 | Journal of Dental Education |
|  | Petre AE et al. | 2023 | Medicina (Lithuania) |
|  | Philip N et al. | 2023 | International Journal of Environmental Research and Public Health |
|  | Pilcher ES | 2001 | European Journal of Dental Education |
|  | Pileggi R and O'Neill PN | 2008 | Journal of Dental Education |
|  | Pinsky HM et al. | 2018 | European Journal of Dental Education |
|  | Plastow K and Gardner S | 2018 | Proceedings of the International Conference on e-Learning, ICEL |
|  | Poblete P et al. | 2020 | Dentistry Journal |
|  | Pogge EK et al. | 2018 | American Journal of Pharmaceutical Education |
|  | Pouhaër M et al. | 2022 | European Journal of Dental Education |
|  | Prabhu S et al. | 2019 | Journal of Dental Education |
|  | Prakash A et al. | 2023 | Journal of Education and Health Promotion |
|  | Prakash K and Prakash R | 2024 | Journal of Dental Education |
|  | Pratheebha C and Jayaraman M | 2022 | Journal of Advanced Pharmaceutical Technology and Research |
|  | Price RB et al. | 2014 | Journal of the American Dental Association |
|  | Quick KK and Blue CM | 2019 | Journal of Dental Education |
|  | Quoß M et al. | 2017 | Zeitschrift fur Evidenz, Fortbildung und Qualitat im Gesundheitswesen |
|  | Qutieshat A et al. | 2022 | International Journal of Dentistry |
|  | Qutieshat A et al. | 2019 | Medical Science Educator |
|  | Rafai N et al. | 2016 | BMC Medical Education |
|  | Rahman A et al. | 2013 | GMS Zeitschrift fur Medizinische Ausbildung |
|  | Raja S et al. | 2015 | Journal of Dental Education |
|  | Rajeh MT et al. | 2020 | Open Dentistry Journal |
|  | Rajeh MT et al. | 2021 | Journal of Dental Education |
|  | Ramachandra SS et al. | 2021 | Journal of Oral Biology and Craniofacial Research |
|  | Rao GKL and Mokhtar N | 2023 | Handbook of Research on Instructional Technologies in Health Education and Allied Disciplines |
|  | Raponi J-M et al. | 2023 | European Journal of Dental Education |
|  | Reeson MG et al. | 2015 | Journal of Dental Education |
|  | Restrepo M et al. | 2024 | European Journal of Dental Education |
|  | Reynolds PA et al. | 2007 | British Dental Journal |
|  | Riaz F et al. | 2015 | Journal of the Pakistan Medical Association |
|  | Rodis OMM and Locsin RC | 2019 | BMC Medical Education |
|  | Roganović J | 2024 | International Dental Journal |
|  | Röhle A et al. | 2021 | GMS Journal for Medical Education |
|  | Romito L et al. | 2014 | Journal of Dental Education |
|  | Romito LM and Eckert GJ | 2011 | Journal of Dental Education |
|  | Rung A et al. | 2014 | JMIR mHealth and uHealth |
|  | Rungnava P et al. | 2018 | ASCILITE - Conference Proceedings - 35th International Conference of Innovation, Practice and Research in the use of Educational Technologies in Tertiary Education: Open Oceans: Learning Without Borders |
|  | Saadaldin SA et al. | 2022 | International Journal of Dentistry |
|  | Sadid-Zadeh R et al. | 2018 | Journal of Dental Education |
|  | Sakka S | 2024 | Journal of Taibah University Medical Sciences |
|  | Salajan F and Prakki A | 2014 | Proceedings of the International Conference on e-Learning, ICEL |
|  | Salajan FD and Mount GJ | 2012 | Journal of Dental Education |
|  | Salehi MM et al. | 2024 | Journal of Dental Education |
|  | Salloum S et al. | 2024 | Proceedings of 2nd International Conference on Advancements in Smart, Secure and Intelligent Computing, ASSIC |
|  | Sangappa SB and Tekian A | 2013 | Journal of Dental Education |
|  | Saqr M et al. | 2020 | BMC Medical Education |
|  | Sarwar H et al. | 2020 | European Journal of Dentistry |
|  | Scally KJ et al. | 2015 | British Dental Journal |
|  | Serrano CM et al. | 2023 | European Journal of Dental Education |
|  | Shah H et al. | 2024 | Journal of the Pakistan Medical Association |
|  | Shah S et al. | 2023 | Pakistan Armed Forces Medical Journal |
|  | Sharif M et al. | 2020 | Pakistan Armed Forces Medical Journal |
|  | Sharma MN et al. | 2022 | International Journal of Early Childhood Special Education |
|  | Sharma P et al. | 2020 | International Journal of Current Research and Review |
|  | Sheikhi M and Mostajabi M | 2024 | Journal of Medical Education Development |
|  | Sheridan RA et al. | 2016 | Journal of Dental Education |
|  | Shigli K et al. | 2017 | Journal of Indian Prosthodontist Society |
|  | Shrivastava KJ et al. | 2022 | European Journal of Dental Education |
|  | Singh R et al. | 2016 | Journal of the Anatomical Society of India |
|  | Sipiyaruk K et al. | 2017 | Technology, Knowledge and Learning |
|  | Sipiyaruk K et al. | 2016 | Lecture Notes of the Institute for Computer Sciences, Social-Informatics and Telecommunications Engineering, LNICST |
|  | Sledge R et al. | 2014 | Journal of Dental Hygiene |
|  | Smith PD and Mays KA | 2019 | Journal of Dental Education |
|  | Sonnenberg LK et al. | 2024 | Learning Environments Research |
|  | Souza FB et al. | 2019 | Journal of Clinical and Diagnostic Research |
|  | Storrs MJ et al. | 2022 | Journal of Dental Education |
|  | Suárez A et al. | 2022 | International Journal of Environmental Research and Public Health |
|  | Suebnukarn S | 2009 | Journal of Dental Education |
|  | Sukotjo C et al. | 2021 | Journal of Dental Education |
|  | Suksudaj N et al. | 2015 | European Journal of Dental Education |
|  | Surya C et al. | 2020 | International Journal of Pharmaceutical Research |
|  | Suyambukesan S and Perumal GCL | 2023 | Malaysian Journal of Medicine and Health Sciences |
|  | Takagi D et al. | 2019 | Educational Gerontology |
|  | Takata M et al. | 2024 | Cureus Journal of Medical Science |
|  | Tanculescu O et al. | 2023 | International Journal of Environmental Research and Public Health |
|  | Tanoubi I et al. | 2022 | European Journal of Investigation in Health, Psychology and Education |
|  | Tawasinchanadech N et al. | 2024 | Journal of Endodontics |
|  | Teparrukkul H et al. | 2024 | Journal of Dental Sciences |
|  | Thammasitboon K et al. | 2007 | Journal of Dental Education |
|  | Thippeswamy HM et al. | 2022 | Journal of Datta Meghe Institute of Medical Sciences University |
|  | Towers A et al. | 2022 | European Journal of Dental Education |
|  | Tricio JA et al. | 2022 | Journal of Dental Education |
|  | Tsang AKL and Walsh LJ | 2010 | European Journal of Dental Education |
|  | Turkyilmaz I et al. | 2019 | Journal of Contemporary Dental Practice |
|  | Urbankova A | 2010 | Journal of Dental Education |
|  | Urbankova A and Engebretson SP | 2011 | Journal of Dental Education |
|  | Vahed A and de Souza FB | 2023 | International Conference on Multidisciplinary Research |
|  | Varghese SS and Aneesa N | 2021 | International Journal of Dentistry and Oral Science |
|  | Vázquez-Rodríguez I et al. | 2020 | Journal of Prosthetic Dentistry |
|  | Victoroff KZ and Hogan S | 2006 | Journal of Dental Education |
|  | Vincent M et al. | 2022 | Journal of Dental Education |
|  | Von Bergmann H et al. | 2017 | Journal of Dental Education |
|  | Vražić D et al. | 2022 | Acta Stomatologica Croatica |
|  | Wadgave U et al. | 2020 | International Journal of Evidence-based Healthcare |
|  | Watanabe M et al. | 2020 | Anatomical Sciences Education |
|  | Welk A et al. | 2005 | European Journal of Dental Education |
|  | Wilkinson DM et al. | 2015 | Journal of Dental Hygiene |
|  | Williamson K et al. | 2023 | Currents in Pharmacy Teaching and Learning |
|  | Woelber JP et al. | 2012 | European Journal of Dental Education |
|  | Wong G et al. | 2020 | Journal of Dental Education |
|  | Wong G et al. | 2019 | European Journal of Dental Education |
|  | Woodman T et al. | 2002 | European Journal of Dental Education |
|  | Wrbas K-T et al. | 2000 | European Journal of Dental Education |
|  | Wright EF and Hendricson WD | 2010 | Journal of Dental Education |
|  | Xiao N et al. | 2021 | Education Sciences |
|  | Yang E et al. | 2023 | Journal of Computing in Higher Education |
|  | Yang Y et al. | 2012 | Knowledge Management and E-Learning |
|  | Zahra D et al. | 2019 | European Journal of Dental Education |
|  | Zhang SY et al. | 2012 | Journal of Dental Education |
|  | Zhang XY et al. | 2023 | JMIR Formative Research |
|  | Zhao R et al. | 2023 | Biochemistry and Molecular Biology Education |
|  | Ziada H et al. | 2024 | Dentistry Journal |
|  | Ziane-Casenave S et al. | 2022 | European Journal of Dental Education |
| No comparison with traditional learning methods | Abd-Shukor SN et al. | 2021 | European Journal of Dental Education |
|  | Al-Saud LM et al. | 2017 | European Journal of Dental Education |
|  | Amer RS et al. | 2011 | Journal of Dental Education |
|  | Bhandary M et al. | 2024 | Journal of Dental Education |
|  | Borit M and Stangvaltaite-Mouhat L | 2020 | European Journal of Dental Education |
|  | Chi DL et al. | 2014 | Journal of Dental Education |
|  | Dalgalı P et al. | 2024 | Cleft Palate Craniofacial Journal |
|  | Fidan M and Fidan M | 2024 | Journal of Computer Assisted Learning |
|  | Gorucu-Coskuner H et al. | 2020 | Journal of Dental Education |
|  | Haley CM et al. | 2020 | Journal of Dental Education |
|  | Hefnawi O et al. | 2022 | Journal of the California Dental Association |
|  | Hobo K et al. | 2017 | Journal of Medical and Dental Sciences |
|  | Karaca O et al. | 2024 | European Journal of Dental Education |
|  | Kavadella A et al. | 2024 | JMIR Medical Education |
|  | Khubchandani M et al. | 2022 | Cureus Journal of Medical Science |
|  | Kieser J et al. | 2008 | Anatomical Sciences Education |
|  | Li ZZ et al. | 2024 | Scientific Reports |
|  | Li G et al. | 2024 | Work |
|  | Li L et al. | 2024 | Heliyon |
|  | Lim J et al. | 2022 | BMC Medical Education |
|  | Lim J et al. | 2019 | BMC Medical Education |
|  | Lohman MC and Finkelstein M | 2002 | European Journal of Dental Education |
|  | Lohman MC and Finkelstein M | 2000 | Instructional Science |
|  | Lone M et al. | 2024 | European Journal of Dental Education |
|  | Macluskey M et al. | 2024 | International Journal of Dentistry |
|  | Mardani M et al. | 2020 | Journal of Dental Education |
|  | Marei HF et al. | 2018 | Medical Teacher |
|  | Martínez-Melo K et al. | 2023 | European Journal of Dental Education |
|  | Mattheos N et al. | 2004 | European Journal of Dental Education |
|  | McAlpin E et al. | 2021 | European Journal of Dental Education |
|  | McAlpin E et al. | 2023 | Learning and Instruction |
|  | McCann AL et al. | 2010 | Journal of Dental Education |
|  | McClure AR et al. | 2019 | Journal of Dental Education |
|  | McHarg J et al. | 2012 | European Journal of Dental Education |
|  | Menon RK and Seow LL | 2021 | Healthcare (Switzerland) |
|  | Metz CJ and Metz MJ | 2021 | Advances in Physiology Education |
|  | Miyoshi T et al. | 2017 | Journal of Medical and Dental Sciences |
|  | Mücke K et al. | 2024 | European Journal of Dental Education |
|  | Miller KT et al. | 2007 | American Journal of Orthodontics and Dentofacial Orthopedics |
|  | Mitov G et al. | 2020 | Journal of Dental Education |
|  | Nasseri A et al. | 2024 | Journal of Dental Sciences |
|  | Nishigawa K et al. | 2017 | Journal of Prosthodontic Research |
|  | Pereira AC et al. | 2022 | European Journal of Dental Education |
|  | Reymus M et al. | 2020 | International Endodontic Journal |
|  | Sadhu B et al. | 2023 | World Journal of Dentistry |
|  | Sehic A and Khan Q | 2024 | Dentistry Journal |
|  | Syed SA et al. | 2024 | PLoS ONE |
|  | Tee WX et al. | 2022 | Healthcare (Switzerland) |
|  | Thomas BS et al. | 2019 | Journal of Orofacial Sciences |
|  | Veeraiyan DN et al. | 2022 | International Journal of Environmental Research and Public Health |
|  | Venturin JS et al. | 2013 | Journal of Dental Education |
|  | Wang BY et al. | 2024 | BMC Medical Education |
|  | Wang HY et al. | 2021 | Annals of Translational Medicine |
|  | Yan Y et al. | 2023 | BMC Medical Education |
| No English or Spanish | Ratzmann A et al. | 2013 | GMS Zeitschrift fur Medizinische Ausbildung |
| Non dental students | Athar U et al. | 2023 | Journal of the Pakistan Medical Association |
|  | Abid K et al. | 2010 | Journal of the Pakistan Medical Association |
|  | Akiyama K et al. | 2023 | Journal of Medical and Dental Sciences |
|  | Alobaid MA et al. | 2022 | International Journal of Environmental Research and Public Health |
|  | Alturkustani S et al. | 2024 | Translational Research in Anatomy |
|  | Brame JL et al. | 2012 | Journal of Dental Hygiene |
|  | Canasi DM et al. | 2014 | Journal of Dental Hygiene |
|  | Cheng BSS | 2009 | Journal of Dental Education |
|  | Claiborne DM et al. | 2021 | Journal of Dental Education |
|  | Cobban SJ and Seale LN | 2003 | International journal of dental hygiene |
|  | Croffoot C et al. | 2010 | Journal of Dental Hygiene |
|  | Deshpande P et al. | 2015 | Journal of Evolution of Medical and Dental Sciences |
|  | Duan D et al. | 2024 | Journal of Dental Education |
|  | Farkhondeh A and Geist JR | 2015 | The Journal of the Michigan Dental Association |
|  | Gadbury-Amyot CC et al. | 2009 | Journal of Dental Education |
|  | Garland, K.V. | 2010 | Journal of Dental Education |
|  | Gordy XZ et al. | 2019 | Journal of Dental Education |
|  | Gottlieb R et al. | 2011 | Journal of Dental Education |
|  | Gray MM et al. | 2022 | Journal of Perinatology |
|  | Gupta A et al. | 2022 | Education Research International |
|  | Ha J-E and Choi DY | 2019 | Anatomy and Cell Biology |
|  | Hashmi NR | 2014 | Journal of the College of Physicians and Surgeons Pakistan |
|  | Im J-E et al. | 2023 | BMC Medical Education |
|  | Johnstone-Dodge V et al. | 2014 | Journal of Dental Education |
|  | Kim D-H | 2023 | Clinical Anatomy |
|  | Kolcu MIB et al. | 2020 | Journal of Dental Education |
|  | Koole S et al. | 2015 | Swedish Dental Journal |
|  | Kumar B et al. | 2022 | Rawal Medical Journal |
|  | Lemaster M et al. | 2016 | Journal of Dental Hygiene |
|  | McAndrew M | 2010 | Journal of Dental Education |
|  | Moore TS | 2007 | Journal of Dental Education |
|  | Moreno-López R and Sinclair S | 2020 | European Journal of Dental Education |
|  | Møystad A et al. | 2015 | European Journal of Dental Education |
|  | Nguyen VH et al. | 2024 | Journal of Dental Education |
|  | Oki AS et al. | 2020 | Systematic Reviews in Pharmacy |
|  | Rahaei Z et al. | 2022 | International Journal of Dental Hygiene |
|  | Rathnakar UP et al. | 2011 | Journal of Clinical and Diagnostic Research |
|  | Reynolds PA et al. | 2007 | Acta Stomatologica Croatica |
|  | Wan T et al. | 2024 | Journal of Dental Education |
|  | Zhu L et al. | 2020 | Nurse Education Today |
| Non retrieved / No reply from corresponding author | Allen KL et al. | 2006 | The New York state dental journal |
|  | Alnowaiser HM et al. | 2019 | Indo American Journal of Pharmaceutical Sciences |
|  | Alvarez WS et al. | 2018 | Dilemas Contemporáneos Educacion Politica y Valores |
|  | Cheung GSP et al. | 1992 | Australian Dental Journal |
|  | Clark RD et al. | 1997 | British Journal of Orthodontics |
|  | Diachkova EY et al. | 2018 | Indo American Journal of Pharmaceutical Sciences |
|  | Esan TA and Oziegbe EO | 2015 | African Journal of Medicine and Medical Sciences |
|  | Puskas JC et al. | 1991 | Journal of Dental Education |
|  | Qi S et al. | 2013 | Journal of Dental Education |
|  | Said NAM et al. | 2022 | Journal of International Dental and Medical Research |
|  | Schweitzer KL and Cohen PA | 1987 | Journal of Dental Education |
|  | Söderström T et al. | 2014 | Simulation and Gaming |
|  | Takkunen M et al. | 2011 | Medical Teacher |
| Other type of intervention (No educational methodology / No strict comparison / Global evaluation of preferences) | Alfallaj HA et al. | 2021 | Open Dentistry Journal |
|  | Ali K et al. | 2020 | European Journal of Dental Education |
|  | Ali K et al. | 2022 | European Journal of Dental Education |
|  | Allen KL and Katz RV | 2011 | Journal of Dental Education |
|  | Allers N | 2010 | Journal of Dental Education |
|  | Alsaleh SA et al. | 2024 | Heliyon |
|  | Al-Thomali Y | 2021 | International Journal of Current Research and Review |
|  | Amin M et al. | 2017 | Journal of Dental Education |
|  | Amine M et al. | 2022 | International Journal of Dentistry |
|  | Aragao MGB et al. | 2022 | European Journal of Dental Education |
|  | Armandeh A et al. | 2021 | Journal of Medical Education Development |
|  | Attar RH and Baghdadi ZD | 2015 | European Archives of Paediatric Dentistry |
|  | Bana KFM et al. | 2022 | Journal of the Pakistan Medical Association |
|  | Bordoni N et al. | 2022 | Acta Odontologica Latinoamericana |
|  | Borromeo GL et al. | 2018 | European Journal of Dental Education |
|  | Botelho MG et al. | 2018 | European Journal of Dental Education |
|  | Bush H and Bissell V | 2008 | European Journal of Dental Education |
|  | Caleya AM et al. | 2024 | Education Sciences |
|  | Cao R et al. | 2018 | Journal of Medical and Dental Sciences |
|  | Carrico C et al. | 2024 | Clinical and Experimental Dental Research |
|  | Chacko PM | 2017 | Journal of Evolution of Medical and Dental Sciences |
|  | Chakrabortty K et al. | 2021 | Journal of Prosthetic Dentistry |
|  | Chakravarthy C et al. | 2022 | Journal of Maxillofacial and Oral Surgery |
|  | Chandelkar UK et al. | 2014 | Pharmacologyonline |
|  | Chang TY et al. | 2021 | Journal of Dental Sciences |
|  | Chaturvedi S et al. | 2021 | Journal of Dental Education |
|  | Chowaniec JA et al. | 2018 | Journal of Dental Education |
|  | Clouet R et al. | 2024 | JMIR Research Protocols |
|  | Costa SA et al. | 2021 | Brazilian Journal of Oral Sciences |
|  | Crivello BJ et al. | 2020 | Journal of Dental Education |
|  | Davies BR et al. | 2009 | British Dental Journal |
|  | De Boer IR et al. | 2017 | Simulation in Healthcare |
|  | Deranek K et al. | 2021 | International Journal of Quality and Reliability Management |
|  | Dhaliwal HK et al. | 2015 | Journal of Orthodontics |
|  | Dixon J et al. | 2021 | European Journal of Dental Education |
|  | ElGolli-Bennour E et al. | 2023 | Education and Information Technologies |
|  | Eroǧlu E et al. | 2022 | BioMed Research International |
|  | Evans J et al. | 2015 | British Dental Journal |
|  | Eve EJ et al. | 2014 | Journal of Dental Education |
|  | Fadel HT et al. | 2021 | ICSIT - 12th International Conference on Society and Information Technologies, Proceedings |
|  | Fadillah R et al. | 2024 | Journal of International Dental and Medical Research |
|  | Fahim A et al. | 2021 | BioMed Research International |
|  | Farrukh K et al. | 2023 | Journal of the Pakistan Medical Association |
|  | Fischer K et al. | 2024 | European Journal of Dental Education |
|  | Ganji KK | 2017 | Journal of Dental Education |
|  | Ganji KK et al. | 2022 | BMC Medical Education |
|  | Goob J et al. | 2021 | Journal of Dental Education |
|  | Gottlieb R et al. | 2017 | Journal of Dental Education |
|  | Green TG and Klausner LH | 1984 | Journal of Dental Education |
|  | Greviana N et al. | 2020 | European Journal of Dental Education |
|  | Gross RT et al. | 2023 | Orthodontics and Craniofacial Research |
|  | Gürsoy M et al. | 2018 | European Journal of Dental Education |
|  | Haghparast N et al. | 2011 | Journal of Dental Education |
|  | Hajhamid B and Somogyi-Ganss E | 2021 | Journal of Dental Education |
|  | Hamzenejad S et al. | 2022 | Strides in Development of Medical Education Journal |
|  | Handal B et al. | 2010 | European Journal of Dental Education |
|  | Haroon S et al. | 2015 | Rawal Medical Journal |
|  | Hassan R et al. | 2022 | Frontiers in Psychology |
|  | Hattar S et al. | 2021 | European Journal of Dental Education |
|  | Hattar S et al. | 2021 | BMC Medical Education |
|  | Haupt F and Kanzow P | 2023 | Interactive Journal of Medical Research |
|  | Hendricson W et al. | 2006 | Journal of Dental Education |
|  | Horst JA et al. | 2009 | Journal of Dental Education |
|  | Islam MI et al. | 2022 | International Journal of Environmental Research and Public Health |
|  | Iyer PK and Leelavathi L | 2022 | International Journal of Early Childhood Special Education |
|  | Jackson TH et al. | 2011 | Journal of Dental Education |
|  | Jain A et al. | 2014 | Journal of Clinical and Diagnostic Research |
|  | Javaid Q and Usmani A | 2024 | Journal of the Pakistan Medical Association |
|  | Jayasinghe RM et al. | 2021 | Asia Pacific Scholar |
|  | Jiang Z et al. | 2021 | BMC Oral Health |
|  | Jones TA | 2019 | Journal of Dental Education |
|  | Kalaimani G et al. | 2023 | Cureus Journal of Medical Science |
|  | Kalghatgi S et al. | 2023 | National Journal of Maxillofacial Surgery |
|  | Karkoutly M et al. | 2024 | BMC Oral Health |
|  | Khalid S et al. | 2021 | Pakistan Journal of Medical & Health Sciences |
|  | Khanagar S et al. | 2021 | Medical Science |
|  | Khanagar SB et al. | 2022 | Applied Sciences (Switzerland) |
|  | Kim G et al. | 2019 | Journal of the Korean Physical Society |
|  | Kononets N et al. | 2021 | Journal of Research in Medical and Dental Science |
|  | Koufogiannakis D et al. | 2005 | Health Information and Libraries Journal |
|  | Kovačević M et al. | 2024 | European Journal of Education |
|  | Kozarovska A and Larsson C | 2018 | European Journal of Dental Education |
|  | Krause F et al. | 2017 | Patient Education and Counseling |
|  | Kui A et al. | 2022 | Medicine and Pharmacy Reports |
|  | Kumar AP et al. | 2023 | PLoS ONE |
|  | Kumar V and Gadbury-Amyot CC | 2012 | Journal of Dental Education |
|  | Leisnert L et al. | 2017 | European Journal of Dental Education |
|  | Lin GSS et al. | 2022 | Education Sciences |
|  | Linjawi AI and Alfadda LS | 2018 | Advances in Medical Education and Practice |
|  | Løset IH et al. | 2022 | International Journal of Environmental Research and Public Health |
|  | Louca C et al. | 2024 | Journal of Dentistry |
|  | Lucander H et al. | 2010 | European Journal of Dental Education |
|  | Maheshwari K et al. | 2022 | Journal of Education and Health Promotion |
|  | Maragha T et al. | 2024 | Journal of Dental Education |
|  | Maupome G and Isyutina O | 2013 | Journal of Dental Education |
|  | Miao X et al. | 2023 | Frontiers in Medicine |
|  | Miletic V et al. | 2024 | Journal of Dental Education |
|  | Mills DA et al. | 2017 | Journal of Dental Education |
|  | Mittal P et al. | 2024 | BMC Oral Health |
|  | Moore R et al. | 2021 | Dentistry Journal |
|  | Morgado M et al. | 2021 | Healthcare (Switzerland) |
|  | Mortadi NA et al. | 2020 | Open Dentistry Journal |
|  | Nahidh M et al. | 2023 | Dentistry Journal |
|  | Nanji A et al. | 2023 | Journal of Dental Education |
|  | Nawabi S et al. | 2021 | Pakistan Journal of Medical Sciences |
|  | Nayak UA et al. | 2022 | Asian Journal of Pharmaceutical Research and Health Care |
|  | Omar E | 2017 | Open Dentistry Journal |
|  | Önöral Ö and Kurtulmus-Yilmaz, S | 2020 | Advanced Education |
|  | Orsini CA et al. | 2019 | Journal of Dental Education |
|  | Oxlad M et al. | 2021 | Australian Psychologist |
|  | Özcan C | 2022 | Oral Health and Preventive Dentistry |
|  | Postma TC and White JG | 2017 | European Journal of Dental Education |
|  | Ria S et al. | 2018 | Journal of Dental Education |
|  | Rodrigues GWL et al. | 2022 | Odontology |
|  | Rowan S et al. | 2017 | Journal of Dental Education |
|  | Schweyen R et al. | 2020 | International Journal of Implant Dentistry |
|  | Sekhon TS et al. | 2022 | Przeglad Epidemiologiczny |
|  | Seki N et al. | 2021 | Journal of Medical and Dental Sciences |
|  | Seki N et al. | 2016 | Journal of Medical and Dental Sciences |
|  | Shariati B et al. | 2021 | Canadian Journal of Dental Hygiene |
|  | Singh R et al. | 2016 | Journal of the Anatomical Society of India |
|  | Syed J et al. | 2024 | Work |
|  | Takeuchi H et al. | 2015 | Journal of Dental Education |
|  | Tang L et al. | 2021 | European Journal of Dental Education |
|  | Tebcherany H and Khocht A | 2024 | Journal of Dental Education |
|  | Thosar N et al. | 2023 | Journal of Datta Meghe Institute of Medical Sciences University |
|  | Toema SM et al. | 2024 | Journal of Dental Education |
|  | Tricio JA et al. | 2017 | Journal of Dental Education |
|  | Veremis BM et al. | 2024 | Journal of Dental Education |
|  | Wierinck E et al. | 2006 | Journal of Dentistry |
|  | Wierinck E et al. | 2006 | European Journal of Dental Education |
|  | Winning T et al. | 2018 | European Journal of Dental Education |
|  | Wolgin M et al. | 2018 | International Journal of Computerized Dentistry |
|  | Wolgin M et al. | 2018 | European Journal of Dental Education |
|  | Wu Y-H and Chiang C-P | 2023 | Journal of Dental Sciences |
|  | Yakin M and Linden K | 2021 | Journal of Dental Education |
|  | Yu WQ et al. | 2023 | European Journal of Dental Education |
| Preclinical / Clinical training | Abe S et al. | 2018 | European Journal of Dental Education |
|  | Akaltan KF et al. | 2023 | Journal of Dental Education |
|  | Al-Zain A et al. | 2023 | Advances in Medical Education and Practice |
|  | Al-Zain AO and Al-Osaimi TM | 2021 | Advances in Medical Education and Practice |
|  | Alsalleeh F et al. | 2024 | Applied Sciences (Switzerland) |
|  | Alzahrani AAH et al. | 2019 | Open Dentistry Journal |
|  | Amtha R et al. | 2018 | Journal of International Dental and Medical Research |
|  | Anamali S et al. | 2022 | European Journal of Dental Education |
|  | Anbarasi K et al. | 2016 | Korean Journal of Medical Education |
|  | Andrews E et al. | 2019 | Journal of Dental Education |
|  | Baghdady MT et al. | 2014 | Journal of Dental Education |
|  | Bai X et al. | 2017 | International Dental Journal |
|  | Barry OP and O'Sullivan E | 2017 | European Journal of Dental Education |
|  | Behar-Horenstein LS et al. | 2015 | Journal of Dental Education |
|  | Bissell V et al. | 2003 | British Dental Journal |
|  | Bjelopavlovic M et al. | 2024 | International Journal of Implant Dentistry |
|  | Bock A et al. | 2021 | Oral Surgery, Oral Medicine, Oral Pathology and Oral Radiology |
|  | Bock A et al. | 2021 | BMC Medical Education |
|  | Bock A et al. | 2021 | BMC Medical Education |
|  | Boynton JR et al. | 2007 | Journal of Dental Education |
|  | Boynton JR et al. | 2007 | Journal of Dental Education |
|  | Bukhary DM and Alshali RZ | 2022 | Journal of Dental Education |
|  | Callis AN et al. | 2010 | Journal of Dental Education |
|  | Canarsky NT et al. | 2023 | Journal of Dental Education |
|  | Carbonaro M et al. | 2008 | Medical Teacher |
|  | Carvalho JC et al. | 2022 | Caries Research |
|  | Chang J et al. | 2024 | Journal of Dental Education |
|  | Chen D et al. | 2023 | PeerJ |
|  | Chen Y et al. | 2024 | Journal of Plastic, Reconstructive and Aesthetic Surgery |
|  | Chen Y-L et al. | 2024 | Journal of Dental Sciences |
|  | Chevalier V et al. | 2022 | International Endodontic Journal |
|  | Choi Y et al. | 2024 | BMC Medical Education |
|  | Chu F et al. | 2023 | BMC Medical Education |
|  | Clancy JM et al. | 2002 | Journal of Dental Education |
|  | Clark NP et al. | 2014 | Journal of Dental Education |
|  | Collaço E et al. | 2021 | Journal of Dental Education |
|  | Corte-Real A et al. | 2021 | Anatomical Sciences Education |
|  | Dantas AK et al. | 2010 | Journal of Dental Education |
|  | Daud A et al. | 2023 | BMC Medical Education |
|  | de Carvalho Rocha B et al. | 2021 | Dentomaxillofacial Radiology |
|  | Dehurtevent M et al. | 2023 | European Journal of Dental Education |
|  | Du G-F et al. | 2013 | European Journal of Dental Education |
|  | Eitner S et al. | 2008 | European Journal of Dental Education |
|  | El Tantawi MMA et al. | 2013 | Journal of Dental Education |
|  | Elashvili A et al. | 2008 | Journal of Dental Education |
|  | Farokhi MR et al. | 2023 | Journal of Dental Education |
|  | Faust AM et al. | 2021 | Journal of Dental Education |
|  | Feil PH and Reed T | 1988 | Journal of dental education |
|  | Fischer K et al. | 2024 | European Journal of Dental Education |
|  | Fu J et al. | 2024 | BMC Oral Health |
|  | Garcia-Blanco M et al. | 2024 | Journal of Dental Education |
|  | Golshah A et al. | 2020 | BMC Medical Education |
|  | Gratton DG et al. | 2016 | Journal of Dental Education |
|  | Hamama H et al. | 2024 | BMC Medical Education |
|  | Hannig A et al. | 2013 | Journal of Educational Computing Research |
|  | Hildebrandt GH and Belmont MA | 2018 | Journal of Dental Education |
|  | Hu J et al. | 2009 | Advances in Health Sciences Education |
|  | Hysi D et al. | 2018 | Acta Stomatologica Croatica |
|  | Iqbal A et al. | 2022 | International Journal of Environmental Research and Public Health |
|  | Jabbour Z and Tran M | 2023 | European Journal of Dental Education |
|  | Janda MS et al. | 2004 | European Journal of Dental Education |
|  | Jeyapalan K et al. | 2016 | Journal of Clinical and Diagnostic Research |
|  | Kalwitzki M et al. | 2010 | European Journal of Dental Education |
|  | Kalwitzki M et al. | 2011 | Journal of Dental Education |
|  | Karl E et al. | 2022 | European Journal of Dental Education |
|  | Kenny KP et al. | 2018 | European Journal of Dental Education |
|  | Khalid S et al. | 2019 | Pakistan Journal of Medical and Health Sciences |
|  | Khattak O et al. | 2022 | Healthcare (Switzerland) |
|  | Kikuchi H et al. | 2013 | Journal of Dental Education |
|  | Knezevic MJ et al. | 2024 | Journal of Clinical Medicine |
|  | Komolpis R and Johnson RA | 2002 | Journal of Dental Education |
|  | König J et al. | 2002 | European Journal of Dental Education |
|  | Koo S et al. | 2015 | Journal of Investigative and Clinical Dentistry |
|  | Kwon SR et al. | 2015 | Journal of Dental Education |
|  | Landes CA et al. | 2014 | Journal of Cranio-Maxillofacial Surgery |
|  | Lau MN et al. | 2021 | PLoS ONE |
|  | Lau MN et al. | 2022 | Journal of Dental Education |
|  | LeBlanc VR et al. | 2004 | Journal of Dental Education |
|  | Lee JS et al. | 2015 | Journal of Dental Education |
|  | Leitmann A et al. | 2020 | BMC Oral Health |
|  | Li L et al. | 2021 | Journal of Medical Internet Research |
|  | Liu K et al. | 2024 | JMIR Serious Games |
|  | Liu L et al. | 2018 | European Journal of Dental Education |
|  | Liu X et al. | 2019 | European Journal of Dental Education |
|  | Lluch AM et al. | 2021 | Dentistry Journal |
|  | López-Cabrera C et al. | 2017 | European Journal of Dental Education |
|  | Lowe CI et al. | 2001 | Journal of Orthodontics |
|  | Lu J et al. | 2022 | BMC Medical Education |
|  | Ludwig B et al. | 2016 | European Journal of Dental Education |
|  | Lugassy D et al. | 2021 | Anatomical Sciences Education |
|  | Ma L et al. | 2024 | Dentistry Journal |
|  | Maggio MP et al. | 2011 | Quintessence International |
|  | Mahrous A et al. | 2023 | Journal of Dental Education |
|  | Mansoory et al. | 2022 | BMC Medical Education |
|  | Marty M et al. | 2019 | European Journal of Dental Education |
|  | McAlpin E et al. | 2022 | European Journal of Dental Education |
|  | McKenzie CT et al. | 2019 | Journal of Dental Education |
|  | McKenzie CT et al. | 2017 | Journal of Dental Education |
|  | Mills DA et al. | 2023 | MedEdPORTAL The Journal of Teaching and Learning Resources |
|  | Mittal P et al. | 2024 | Technology and Health Care |
|  | Mladenovic R et al. | 2022 | BMC Oral Health |
|  | Mladenović R et al. | 2020 | Vojnosanitetski Pregled |
|  | Montero J et al. | 2018 | Journal of Dental Education |
|  | Morales-Vadillo R et al. | 2019 | General Dentistry |
|  | Nikzad S et al. | 2012 | Journal of Dental Education |
|  | Nilsson TA et al. | 2007 | Simulation in Healthcare |
|  | Olms C et al. | 2017 | Annals of Anatomy |
|  | Packer ME et al. | 2001 | European Journal of Dental Education |
|  | Patel SA et al. | 2020 | Journal of Dental Education |
|  | Porcherot A et al. | 2024 | European Journal of Dental Education |
|  | Postma TC and White JG | 2017 | European Journal of Dental Education |
|  | Postma TC and White JG | 2016 | European Journal of Dental Education |
|  | Prasad S and Bansal N | 2017 | Journal of Dental Education |
|  | Pulijala Y et al. | 2018 | Journal of Oral and Maxillofacial Surgery |
|  | Puranik CP et al. | 2022 | Journal of Dental Education |
|  | Quinn F et al. | 2003 | European Journal of Dental Education |
|  | Quinn F et al. | 2003 | European Journal of Dental Education |
|  | Qutieshat AS et al. | 2020 | Journal of Dental Education |
|  | Ramlogan S et al. | 2014 | European Journal of Dental Education |
|  | Rampf S et al. | 2024 | European Journal of Dental Education |
|  | Reddington AR and Weir SO | 2024 | Journal of Dental Education |
|  | Reed SG et al. | 2015 | Journal of Dental Education |
|  | Reissmann DR et al. | 2015 | Journal of Dental Education |
|  | Reyes-Acuca MJ et al. | 2020 | European Journal of Dental Education |
|  | Reymus M et al. | 2018 | International Endodontic Journal |
|  | Rich SK et al. | 2005 | Journal of Dental Education |
|  | Rosa QF et al. | 2013 | European Journal of Dental Education |
|  | Roy E et al. | 2018 | European Journal of Dental Education |
|  | Rystedt H et al. | 2013 | Journal of Dental Education |
|  | Sadid-Zadeh R et al. | 2019 | Journal of Dental Education |
|  | Saheb SAK et al. | 2024 | Journal of Dental Education |
|  | Samuelson DB et al. | 2017 | Journal of Dental Education |
|  | San Diego JP et al. | 2022 | Dentistry Journal |
|  | Santhakumar M and Vidhya R | 2021 | Journal of Indian Society of Pedodontics and Preventive Dentistry |
|  | Savoldi F et al. | 2021 | Anatomical Sciences Education |
|  | Schlafer S et al. | 2021 | Journal of Dental Education |
|  | Schorn-Borgmann S et al. | 2015 | Advances in Medical Education and Practice |
|  | Schuster GM et al. | 2017 | Journal of Dental Education |
|  | Schwindling FS et al. | 2015 | Journal of Dental Education |
|  | Seidel M et al. | 2020 | BMC Oral Health |
|  | Seifert LB et al. | 2020 | European Journal of Dental Education |
|  | Seifert LB et al. | 2019 | BMC Medical Education |
|  | Sennhenn-Kirchner S et al. | 2018 | European Journal of Dental Education |
|  | Sezer B | 2023 | European Journal of Dental Education |
|  | Shaheen MY et al. | 2023 | Cureus Journal of Medical Science |
|  | Sheng J et al. | 2022 | BMC Medical Education |
|  | Siegel SC et al. | 2019 | Journal of Dental Education |
|  | Singhania A et al. | 2024 | Journal of Datta Meghe Institute of Medical Sciences University |
|  | Singleton JA et al. | 2014 | Journal of Dental Education |
|  | Sivarajan S et al. | 2021 | BMC Medical Education |
|  | Sjöström M and Brundin M | 2021 | Dentistry Journal |
|  | Sonkaya E and Kürklü ZGB | 2024 | European Journal of Dental Education |
|  | Stoilov M et al. | 2021 | International Journal of Environmental Research and Public Health |
|  | Suebnukarn S et al. | 2010 | Journal of Dental Education |
|  | Suebnukarn S et al. | 2011 | International Endodontic Journal |
|  | Tahami H et al. | 2024 | Journal of Dental Education |
|  | Tak N-Y et al. | 2023 | European Journal of Dental Education |
|  | Tan YY et al. | 2022 | European Journal of Dental Education |
|  | Tani Botticelli A et al. | 2005 | International Journal of Computerized Dentistry |
|  | Tauböck TT et al. | 2020 | Applied Sciences (Switzerland) |
|  | Taysi AE et al. | 2024 | BMC Medical Education |
|  | Terry A et al. | 2021 | European Journal of Dental Education |
|  | Thelen RM et al. | 2023 | Journal of Dental Education |
|  | Tiu J et al. | 2016 | Journal of Dental Education |
|  | Tricio JA et al. | 2024 | Journal of Dental Education |
|  | Tubelo RA et al. | 2015 | International Journal of Medical Informatics |
|  | Uppgaard R et al. | 2023 | Journal of Dental Education |
|  | Van Der Molen HT et al. | 2004 | British Dental Journal |
|  | Varghese AS et al. | 2024 | Journal of Education and Health Promotion |
|  | Vasil'ev Y et al. | 2022 | Dentistry Journal |
|  | Vincent M et al. | 2020 | Journal of Dental Education |
|  | Wang M et al. | 2024 | BMC Medical Education |
|  | Wang P et al. | 2024 | European Journal of Dental Education |
|  | Wenz H-J et al. | 2014 | European Journal of Dental Education |
|  | Wenzel A et al. | 2020 | Dentomaxillofacial Radiology |
|  | Wierinck E et al. | 2005 | European Journal of Dental Education |
|  | Wu S et al. | 2024 | International Dental Journal |
|  | Wu T et al. | 2024 | BMC Medical Education |
|  | Yang X et al. | 2024 | British Journal of Hospital Medicine |
|  | Yang Y et al. | 2022 | BMC Medical Education |
|  | Yoshida S et al. | 2022 | Journal of Dental Education |
|  | Zahl DA et al. | 2018 | European Journal of Dental Education |
|  | Zain E et al. | 2024 | European Journal of Dental Education |
|  | Zhan Y et al. | 2021 | Journal of Dental Education |
|  | Zhang B et al. | 2020 | BMC Medical Education |
|  | Zhang J et al. | 2021 | BMC Medical Education |
|  | Zhong XY et al. | 2024 | European Journal of Dental Education |
|  | Zhou Y et al. | 2022 | BMC Medical Education |
| Prior to study period | Botelho MG | 1999 | British Dental Journal |
|  | Brickley MR et al. | 1995 | Medical Education |
|  | Chen MS et al. | 1998 | British journal of orthodontics |
|  | Engel FE and Hendricson WD | 1994 | Journal of Dental Education |
|  | Fouad AF and Burleson JA | 1997 | Journal of Dental Education |
|  | Gershen JA | 1978 | Journal of Dental Education |
|  | Gershen JA and Jedrychowski JR | 1979 | Journal of Dental Education |
|  | Hobson RS et al. | 1998 | European Journal of Dental Education |
|  | Houlihan PA et al. | 1992 | Journal of Computer-Based Instruction |
|  | Hutton Jr JG et al. | 1982 | Journal of Dental Education |
|  | Irvine NR and Moore RN | 1986 | Journal of Dental Education |
|  | Johnson JA and Kopp KC | 1996 | Journal of Dental Education |
|  | Joorabchi B | 1979 | Medical Education |
|  | Kassebaum DK et al. | 1991 | Journal of Dental Education |
|  | Kindelan J et al. | 1997 | European Journal of Dental Education |
|  | Kleffner JH and Dadian T | 1997 | Journal of Dental Education |
|  | Last KS et al. | 2001 | European Journal of Dental Education |
|  | Lee MM et al. | 1981 | Journal of Dental Education |
|  | Login GR et al. | 1997 | Journal of Dental Education |
|  | Long NK et al. | 1997 | Operative Dentistry |
|  | Lum-Peng L and Ai-Yen C | 1999 | European Journal of Dental Education |
|  | Manogue M et al. | 1999 | International Endodontic Journal |
|  | Morgan CH et al. | 1988 | Applied Cognitive Psychology |
|  | Mulligan R and Wood GJ | 1993 | Journal of Dental Education |
|  | Oesterle LJ and Shellhart WC | 1998 | European Journal of Dental Education |
|  | Pau AKH et al. | 1999 | European Journal of Dental Education |
|  | Russell MD et al. | 1996 | Journal of Dentistry |
|  | Shugars DA et al. | 1979 | Journal of Dental Education |
|  | Tomar SL et al. | 1998 | Journal of Cancer Education |
|  | Vickers JD | 1990 | Journal of Dental Education |
|  | Wetherell J et al. | 1999 | European Journal of Dental Education |
| Sample size ≤ 6 per cohort | Reynolds PA et al. | 2007 | British Dental Journal |
| Systematic review / other type of review | Acosta RAM et al. | 2017 | Medisur Revista de Ciencias Medicas de Cienfuegos |
|  | Aljabr A | 2021 | Open Dentistry Journal |
|  | Alrahlah A | 2016 | Saudi Dental Journal |
|  | Amos KJ et al. | 2015 | Journal of Dental Education |
|  | Apel Z et al. | 2024 | Journal of Dental Education |
|  | Araujo-Cuauro JC | 2022 | Acta Bioclinica |
|  | Arsiwala-Scheppach LT et al. | 2023 | Journal of Clinical Medicine |
|  | Athanasiou, AE | 2024 | Seminars in Orthodontics |
|  | Bashary NZ and Levine MH | 2024 | Journal of Dental Education |
|  | Bassir SH et al. | 2014 | Journal of Dental Education |
|  | Berry MCD et al. | 2020 | Journal of Dental Education |
|  | Bhat GS et al. | 2021 | Gulhane Medical Journal |
|  | Botelho MG et al. | 2019 | Dentomaxillofacial Radiology |
|  | Burkert V et al. | 2021 | Folia Medica |
|  | Calleja JL et al. | 2020 | Medwave |
|  | de Mendonça BFS et al. | 2024 | Journal of Dental Education |
|  | Dobroś K, Hajto-Bryk J, Zarzecka J | 2023 | European Journal of Dental Education |
|  | Dong H et al. | 2022 | BMJ Open |
|  | Doran GA | 2000 | Journal of the South African Dental Association |
|  | Doran GA | 2000 | Journal of the South African Dental Association |
|  | Dsouza TS et al. | 2022 | Journal of Health and Allied Sciences Un |
|  | Dzyuba N et al. | 2022 | European Journal of Dental Education |
|  | Ebrahimi M et al. | 2023 | Journal of Medical Education Development |
|  | Elemam RF et al. | 2022 | Egyptian Journal of Hospital Medicine |
|  | Gandedkar NH et al. | 2021 | Seminars in Orthodontics |
|  | Garg M et al. | 2022 | British Journal of Oral and Maxillofacial Surgery |
|  | Ghiabi E and Taylor KL | 2010 | Journal of Dental Education |
|  | Gianoni-Capenakas S et al. | 2019 | Journal of Dental Education |
|  | Gönülol N and Kalyoncuoglu E | 2021 | Journal of Experimental and Clinical Medicine (Turkey) |
|  | Hadjichristou C et al. | 2024 | Journal of Dental Education |
|  | Hew KF and Lo CK | 2018 | BMC Medical Education |
|  | Higgins D et al. | 2020 | MedEdPublish (2016) |
|  | Huang B et al. | 2013 | Journal of Dental Education |
|  | Huang Y et al. | 2023 | European Journal of Dental Education |
|  | James Trill B et al. | 2024 | British Dental Journal |
|  | Jin J and Bridges SM | 2014 | Journal of Medical Internet Research |
|  | Kachabian S et al. | 2024 | Evidence-Based Dentistry |
|  | Kary AL et al. | 2018 | Journal of Dental Education |
|  | Khalaf K et al. | 2020 | BMC Medical Education |
|  | Kilgour JM et al. | 2016 | Teaching and Learning in Medicine |
|  | Koolivand H et al. | 2024 | BMC Medical Education |
|  | Kumar A | 2017 | APOS Trends in Orthodontics |
|  | Li Y et al. | 2021 | Journal of Medical Internet Research |
|  | Luke AM et al. | 2021 | BioMed Research International |
|  | Madiyal A et al. | 2021 | Journal of Health and Allied Sciences Un |
|  | Martins JCS et al. | 2022 | Medical Science Educator |
|  | Mattheos N et al. | 2008 | European Journal of Dental Education |
|  | McGleenon EL and Morison S | 2021 | British Dental Journal |
|  | Nagendrababu V et al. | 2019 | International Endodontic Journal |
|  | Najminouri F | 2021 | Journal of Oral Health and Oral Epidemiology |
|  | Overskott HL et al. | 2024 | Dentistry Journal |
|  | Pang X et al. | 2024 | International Dental Journal |
|  | Patil S et al. | 2023 | BMC Oral Health |
|  | Perez A et al. | 2023 | PLoS ONE |
|  | Polyzois I et al. | 2010 | European Journal of Dental Education |
|  | Preshaw PM et al. | 2024 | Journal of Clinical Periodontology |
|  | Prosser M | 2004 | European Journal of Dental Education |
|  | Prosser M and Sze D | 2014 | Clinical Linguistics and Phonetics |
|  | Rao GKL et al. | 2020 | Education and Information Technologies |
|  | Saif A and Umar I | 2021 | Lecture Notes on Data Engineering and Communications Technologies |
|  | Santos GNM et al. | 2016 | Journal of Dental Education |
|  | Shetty S et al. | 2019 | Brazilian Dental Science |
|  | Sipiyaruk K et al. | 2021 | Computers |
|  | Sipiyaruk K et al. | 2023 | Dental Press Journal of Orthodontics |
|  | Tahir W et al. | 2023 | Liaquat National Journal of Primary Care |
|  | Towers A et al. | 2019 | British Dental Journal |
|  | Tsuruta J | 2023 | European Journal of Dental Education |
|  | Turchiello RZ et al. | 2019 | International Journal of Paediatric Dentistry |
|  | Uoshima K et al. | 2021 | Japanese Dental Science Review |
|  | Vanka A et al. | 2020 | European Journal of Dental Education |
|  | Walinski CJ et al. | 2023 | Journal of Dental Education |
|  | Wang J et al. | 2022 | Food Science and Technology (Brazil) |
|  | Winning T and Townsend G | 2007 | Australian Dental Journal |
|  | Woldt JL and Nenad MW | 2021 | Journal of Dental Education |
|  | Zahra FS and Dunton K | 2017 | British Dental Journal |
